# S100A14: A novel negative regulator of cancer stemness and immune evasion by inhibiting STAT3‐mediated programmed death‐ligand 1 expression in colorectal cancer

**DOI:** 10.1002/ctm2.986

**Published:** 2022-07-20

**Authors:** Hye‐Young Min, Jaebeom Cho, Jeong Yeon Sim, Hye‐Jin Boo, Ji‐Sun Lee, Seon‐Boon Lee, Young‐Jin Lee, Sung Joo Kim, Kyu‐Pyo Kim, In‐Ja Park, Seung‐Mo Hong, Xue‐Li Zhang, Zhi‐Gang Zhang, Rang‐Woon Park, Ho‐Young Lee

**Affiliations:** ^1^ Creative Research Initiative Center for Concurrent Control of Emphysema and Lung Cancer, College of Pharmacy Seoul National University Seoul Republic of Korea; ^2^ College of Pharmacy and Research Institute of Pharmaceutical Sciences Seoul National University Seoul Republic of Korea; ^3^ Department of Biochemistry and Cell Biology, School of Medicine, Cell & Matrix Research Institute Kyungpook National University Daegu Republic of Korea; ^4^ Department of Pathology, Asan Medical Center University of Ulsan College of Medicine Seoul Republic of Korea; ^5^ Department of Oncology, Asan Medical Center University of Ulsan College of Medicine Seoul Republic of Korea; ^6^ Department of Surgery, Asan Medical Center University of Ulsan College of Medicine Seoul Republic of Korea; ^7^ State Key Laboratory of Oncogenes and Related Genes, Shanghai Cancer Institute, Ren Ji Hospital, School of Medicine Shanghai Jiao Tong University Shanghai P.R. China

**Keywords:** cancer stem‐like cells, colorectal cancer, PD‐L1, STAT3, S100A14

## Abstract

**Background:**

Programmed death‐ligand 1 (PD‐L1) has functional roles in cancer stem‐like cell (CSC) phenotypes and chemoresistance besides immune evasion. Chemotherapy is a common treatment choice for colorectal cancer (CRC) patients; however, chemoresistance limits its effectiveness of treatment.

**Methods:**

We examined the role of S100A14 (SA14) in CRC by adopting PD‐L1^high^ subpopulations within CRC cell lines and patient tumours, by establishing PD‐L1^high^ chemoresistant CRC sublines through prolonged exposure to 5‐fluorouracil/oxaliplatin‐based chemotherapy in vitro and in vivo, and by analysing a public database.

**Results:**

We identified a novel function of SA14 as a regulator of immune surveillance, major CSC phenotypes, and survival capacity under hostile microenvironments, including those harbouring chemotherapeutics, and as a prognostic biomarker in CRC. Mechanistically, SA14 inhibits PD‐L1 expression by directly interacting with signal transducer and activator of transcription 3 (STAT3) and inducing its proteasome‐mediated degradation. While gain‐of‐SA14 causes loss of PD‐L1 expression and tumourigenic potential and sensitisation to chemotherapy‐induced apoptosis in chemoresistant CRC cells, loss‐of‐SA14 causes increases in PD‐L1 expression, tumourigenic potential, and chemoresistance in vitro and in vivo. We further show that a combinatorial treatment with chemotherapy and recombinant SA14 protein effectively induces apoptosis in PD‐L1^high^ chemoresistant CRC cells.

**Conclusions:**

Our results suggest that SA14‐based therapy is an effective strategy to prevent tumour progression and that SA14 is a predictive biomarker for anti‐PD‐L1 immunotherapy and chemotherapy in combination.

## Graphical abstract

- S100A14 expression is downregulated in PD‐L1^high^ colorectal cancer cells, enabling them to have CSC‐like phenotypes and immune‐suppressive capacities.
- S100A14 inhibits PD‐L1 expression by directly interacting with STAT3 and inducing its proteasomal degradation.
- S100A14 is a potential marker for predicting prognosis and the antitumor response to PD‐L1 blockade and chemotherapy in colorectal cancer.

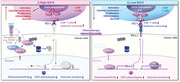

## 1 BACKGROUND

Despite significant breakthroughs in early detection and anticancer treatment, colorectal cancer (CRC) is one of the most lethal malignancy.^1^, ^2^ Currently, conventional chemotherapeutic regimens like 5‐fluorouracil (5‐FU) in combination with oxaliplatin (Oxa, FOLFOX) represent the standard first‐line treatment for CRC, especially CRC patients with metastatic tumours.^3^, ^4^, ^5^ However, the response rate to the FOLFOX‐based chemotherapies in CRC is only 10%–15%.^6^ Recently, the programmed cell death protein 1 (PD‐1)‐targeting immune checkpoint inhibitors (ICIs; e.g., nivolumab and pembrolizumab) have shown significant efficacy among some patients with CRC.^3^, ^5^, ^7^, ^8^ Pembrolizumab was licensed by the US Food and Drug Administration for the treatment of individuals with microsatellite instability (MSH)^high^/deficient mismatch repair (dMMR) CRC who progressed after 5‐FU‐ or Oxa‐based chemotherapy.^8^, ^9^ Several clinical trials have been underway to assess the efficacy of combining ICIs with chemotherapy.^7^, ^10^ However, only a minority of patients with MSH^high^/dMMR CRC exhibited promising response to ICIs.^7^ Therefore, understanding the mechanism underlying programmed death‐ligand 1 (PD‐L1) expression and identification of markers enabling better prediction of the benefit from targeting the PD‐L1/PD‐1 axis could provide innovative treatment regimens for CRC patients.

While chemotherapy is known to target rapidly proliferating cancer cells,^11^ several chemotherapeutic agents, including 5‐FU and Oxa, are known to activate the immune system by depleting myeloid‐derived suppressor cells, by inducing immunogenic cell death‐associated release of damage‐associated molecular pattern molecules, and/or by regulating PD‐L1.^10^, ^12^, ^13^ PD‐L1 is one of two PD‐1 receptor ligands and belongs to the B7 family of T‐cell coregulatory molecules.^14^ The binding of PD‐L1 to PD‐1 prevents the proliferation and activation of T cells, thereby inducing T‐cell apoptosis and immune evasion.^13^ PD‐L1 has also been proposed to have tumour‐intrinsic roles in the survival and progression of cancer cells by stimulating their epithelial‐mesenchymal transition (EMT) and cancer stem‐like cell (CSC) properties.^14^, ^15^ Certain types of cancer cells were found to become resistant to 5‐FU or Oxa through increased PD‐L1 expression.^16^, ^17^ Studies have shown that PD‐L1 is regulated genetically, transcriptionally and posttranslationally.^18^ However, the molecular processes behind PD‐L1 expression regulation in chemoresistant cancer cells remain unclear.

The S100 protein family is the largest subgroup of calcium‐binding EF‐hand‐type low‐molecular‐weight proteins^19^, ^20^ and exerts several intracellular functions responsible for cell proliferation, apoptosis and motility, all of which are implicated in tumour development and progression.^21^ S100s bind to a number of proteins on the cell surface, including receptor for advanced glycation end products (RAGEs), toll‐like receptor 4, human epidermal growth factor receptor 2 (HER2) and heparan sulphate proteoglycan, and regulate various signal transduction pathways, such as phosphoinositide 3‐kinase/Akt, mitogen‐activated protein kinases and Cdc42/Rac.^22^, ^23^, ^24^ S100A14 (hereafter SA14) was recently identified as an S100 member protein.^25^ Studies have shown elevated expression in breast and uterine cancers but decreased expression in colon, kidney and rectal cancers.^25^ Elevated SA14 expression is related to poor clinical outcomes in individuals with ovarian, breast and cervical cancer,^26^ while decreased expression was associated with poor prognosis and disease progression in patients with colorectal, small intestine, gastric and oesophageal cancers.^26^, ^27^, ^28^ SA14 has also shown cancer type‐dependent differential action.^25^ For instance, SA14 inhibits proliferation and invasion of oral squamous carcinoma.^29^, ^30^ In contrast, SA14 stimulates proliferation of breast cancer cells by interacting with HER2,^31^ induces gemcitabine resistance in pancreatic cancer cells,^32^ and engenders motility and invasion of breast cancer cells.^33^ SA14 has also been involved in the differentiation of oesophageal and gastric cancers^34^, ^35^ and regulation of immunosurveillance.^36^ However, the role of SA14 in CRC is mostly unclear.

In this study, we aimed to examine the association of PD‐L1 with acquiring CSC phenotypes and chemoresistance, explore the mechanisms by which PD‐L1 expression is regulated, and identify biomarkers that predict the effectiveness of ICI‐based therapeutic regimens in CRC. Here, we demonstrate a novel role of SA14 that acts as a regulator of stemness and immune evasive capacity through disruption of signal transducer and activator of transcription 3 (STAT3)‐mediated PD‐L1 expression.

## 2 METHODS

### 2.1 Reagents

Table S1 contains detailed information on the primary and secondary antibodies (Abs) utilised, such as catalogue number, supplier and dilution ratio (or concentration) for each application. A mammalian expression vector carrying SA14 was purchased from Origene (catalogue no. RG202590; Rockville, MA, USA). A mammalian expression vector carrying the constitutively active STAT3 mutant (STAT3 Y705D) was provided as described in a previous report.^37^

### 2.2 Cell culture

Human CRC cells (SW480, HT‐29, HCT‐15 and HCT116) were kindly provided by Dr. Sang Kook Lee (Seoul National University, Seoul, Republic of Korea). CT26 cells were purchased from the Korean Cell Line Bank (Seoul, Republic of Korea). These cells were grown in Roswell Park Memorial Institute (RPMI) 1640 supplemented with 10% foetal bovine serum (FBS) and 1× antibiotic‐antimycotic solution (antibiotics, Welgene, Kyeongsan‐si, Republic of Korea). Chemoresistant sublines were produced by over 6 months of continuous treatment with the chemotherapeutic agent. Authentication and verification of human cancer cell lines were performed in 2013, 2016 and 2020 using the AmplFLSTR identifier PCR Amplification Kit (Applied Biosystems, Foster, CA, USA; catalogue no. 4322288). This study utilised mycoplasma‐free cells maintained for less than 3 months.

### 2.3 Animal experiments

We carried out all animal studies in accordance with protocols approved by the Institutional Animal Care and Use Committee of Seoul National University (approval nos. SNU‐201026‐5‐1 and SNU‐211214‐3). Tumour xenograft models were established by subcutaneously inoculating 5‐FU‐resistant HCT116 (HCT/FuR) cells that had been stably transfected with empty vector (EV) or SA14‐overexpressing vector (HCT/FuR‐EV and HCT/FuR‐SA14 cells, respectively; 4 × 10^6^ cells) into the right flanks of nonobese diabetic/severe combined immunodeficiency (NOD/SCID) mice (4–6 weeks old) (Animal Resources Center, Canning Vale, Western Australia, Australia). Tumour size was measured using a caliper for 4 weeks, three times each week.

For xenograft tumours from parental HCT116 (HCT/P) cells transduced with control shRNA (HCT/P‐shCon) or shS100A14 (HCT/P‐shSA14), 3 × 10^6^ cells were subcutaneously inoculated into the right flanks of nude mice (8 weeks old). When tumour volumes reached 50 mm^3^, a combination of chemotherapeutic agents [5‐FU (50 mg/kg, dissolved in 10% dimethyl sulphoxide in phosphate‐buffered saline, PBS) and Oxa (6 mg/kg, dissolved in 0.9% NaCl)] was given intraperitoneally once a week for 3 weeks.

For the allograft experiment, CT26‐EV and CT26‐SA14 cells (1.5 × 10^5^ cells/spot, diluted in PBS) were inoculated subcutaneously into the right flanks of 8‐week‐old female Balb/c mice. When the tumour volume reached 50–100 mm^3^, the mice were grouped at random and intraperitoneally treated with either vehicle, anti‐PD‐L1 antibody (Ab) (BioXCell, Lebanon, NH, USA; 100 μg per mouse), or a combination of chemotherapeutic agents (50 mg/kg 5‐FU and 6 mg/kg Oxa, solvated as described above). Chemotherapeutic agents were administered once a week, and anti‐PD‐L1 Ab was administered twice per week. The growth of the tumour was assessed as described in our previous report.^38^

### 2.4 Analysis of public datasets

A detailed procedure for the analysis of publicly accessible datasets was performed as stated in our previous report^38^. The SA14^high^ and SA14^low^ groups were determined using the median value of the data in each dataset. The highest and lowest 25th percentiles were used to define the SA14^high^ and SA14^low^ groups for Kaplan‒Meier survival analysis. To determine significance, the log‐rank test was performed. The probes utilised for acquiring gene expression levels for each dataset are shown in Table S2.

### 2.5 Immunohistochemistry and immunofluorescence

Immunohistochemistry (IHC) and immunofluorescence analyses were carried out as previously reported.^38^

### 2.6 Flow cytometry

#### 2.6.1 Flow cytometric analysis for determining PD‐L1 levels on the cell membrane in CRC cells

A total of 1 × 10^5^ cells were treated on ice for 15 min with FcR blocking reagent (Miltenyl Biotec, Bergisch Gladbach, Germany) diluted in fluorescence‐activated cell sorting (FACS) buffer (PBS containing 1% bovine serum albumin (BSA), 2 mM ethylenediaminetetraacetic acid (EDTA) and 0.05% sodium azide, 1:50 ratio). Cells were treated on ice for 1 h with mouse immunoglobulin G (IgG) isotype control or anti‐PD‐L1 primary Abs diluted in FACS buffer (BioLegend, San Diego, CA, USA, 1:100 ratio). After washing twice with FACS buffer, the cells were stained on ice for 30 min with secondary Abs conjugated to Alexa Fluor 633 (Thermo Fisher Scientific, Waltham, MA, USA, 1:200 ratio). Cells were examined using a FACSCalibur flow cytometer after being washed twice with FACS buffer (BD Biosciences, San Jose, CA, USA). FlowJo software (BD Biosciences) was used to determine the degree of PD‐L1 positivity.

#### 2.6.2 Isolation of the PD‐L1^high^ and PD‐L1^low^ populations

A Tumour Dissociation Kit (Miltenyl Biotec) was used to separate primary CRC tumour cells from patient‐derived xenograft tumours in accordance with the manufacturer's instructions. Cells were stimulated with interferon‐gamma (IFN‐γ, 10 ng/ml) for 24 h before staining if needed. Primary CRC cells or CRC cell lines were stained with anti‐PD‐L1 Abs diluted in FACS buffer (1:100 ratio), washed twice with FACS buffer, and then stained with phycoerythrin (PE)‐conjugated secondary Abs (Thermo Fisher Scientific, 1:200 ratio). After being washed twice with FACS buffer, a FACS Aria III flow cytometer (BD Biosciences) was used to sort the labelled cells for subsequent in vitro investigations.

#### 2.6.3 Isolation of the CD133^high^CD44^high^ and CD133^low^CD44^low^ populations

CRC cells and primary CRC tumour cells were stained with fluorescein 5‐isothiocyanate (FITC)‐conjugated anti‐CD44 and PE‐conjugated anti‐CD133 Abs diluted in FACS buffer (1:100 ratio) and then sorted with a FACS Aria III flow cytometer after being washed twice with FACS buffer.

#### 2.6.4 Analysis of activated CD8^+^ T lymphocytes using flow cytometry

A CD8^+^ T‐cell isolation kit (Miltenyl Biotec) was used to separate splenic CD8^+^ T cells from the newly isolated spleen of 8‐week‐old male Balb/c mice. CD8^+^ T cells were stimulated for 3 days in RPMI 1640 medium (containing 50 μM β‐mercaptoethanol, antibiotics and 10% FBS) with anti‐CD3 and anti‐CD28 Abs. CT26‐EV and CT26‐SA14 cells (1 × 10^4^ cells/well) were plated in 96‐well plates and incubated for 1 day. After incubation, the cells were cocultured with activated T cells at 2:1 ratio for 12 h (T cells:tumour cells). Before harvest, cells were cultured for 6 h in the presence of brefeldin A (5 μg/ml, BioLegend). Following coculture with cancer cells, collected T lymphocytes were fixed and permeabilised according to the manufacturer's instructions using the Cytofix/Cytoperm fixation/permeabilisation solution kit (BD Bioscience). Thereafter, the cells were stained for 30 min on ice with a FACS buffer‐diluted PE‐conjugated anti‐IFN‐γ Ab (BioLegend) (1:100 ratio). Next, the cells were washed twice with FACS buffer and flow cytometrically examined using a FACSCalibur flow cytometer. The IFN‐γ^+^CD8^+^ T‐cell level was calculated using the FlowJo program.

#### 2.6.5 Analysis of tumour‐infiltrating CD8^+^ T lymphocytes using flow cytometry

CT26 allograft tumours were extracted from killed mice and digested using the Tumour Dissociation Kit (Miltenyl Biotec) as directed by the manufacturer. To prevent nonspecific Ab binding to FcR‐expressing tumour‐infiltrated immune cells such as B cells and macrophages, single‐cell suspensions from tumours were incubated on ice for 10 min with TruStain fcX (BioLegend) diluted in a FACS buffer (BioLegend, 1:50 ratio) before being stained with PerCP/Cy5.5‐conjugated anti‐CD3e (BioGems, Westlake Village, CA, USA, 1:200 ratio) and allophycocyanin‐conjugated anti‐CD8 Abs (BioGems, 1:200 ratio) on ice for 30 min. Cells were examined using a FACSCalibur flow cytometer after being washed twice with FACS buffer. The CD8^+^ T‐cell count in the tumour was assessed using the FlowJo program.

### 2.7 Cell viability, sphere formation, anchorage‐dependent colony formation and anchorage‐independent colony formation assays

Assays for testing cell viability, sphere formation, anchorage‐dependent (AD) colony formation and anchorage‐independent (AID) colony formation were carried out as previously reported.^38^

### 2.8 Staining with Hoechst 33342

Following drug treatment, the cells were treated with Hoechst 33342 (20 μM, Thermo Fisher Scientific) for 30 min. A fluorescent microscope was used to examine the cells. Cells with fragmented, deteriorated or condensed nuclei were counted and designated apoptotic.

### 2.9 Western blot analysis and real‐time PCR

Western blot (WB) and real‐time PCR analyses were carried out as previously reported.^38^ The sequences of the primers utilised in the PCR experiments are listed in Table S3.

### 2.10 Cloning, expression and purification of recombinant proteins

The glutathione‐S‐transferase (GST) and GST‐tagged full‐length STAT3 plasmid constructs were created by cloning them into the BamHI/XhoI site of pGEX‐4T‐2 (GE Healthcare Life Sciences, Chicago, IL, USA). For the His‐tagged bacterial SA14 protein, the SA14 coding sequence was subcloned into the pET28a vector by using EcoRI/SalI restriction enzymes. Clones encoding GST alone or GST‐STAT3 were expressed in *Escherichia coli* BL21 by overnight culture at 25°C in Luria‐Bertani (LB) medium supplemented with 1 mM isopropyl‐β‐D‐1‐thiogalactopyranoside (IPTG) and purified using Glutathione Sepharose 4B (GE Healthcare). His‐tagged SA14 protein was expression in *E. coli* BL21 by 6 h of incubation at 30°C in LB medium. After bacterial cell lysis, histidine‐tagged protein was captured by nickel‐nitrilotriacetic acid (Ni‐NTA) beads.

### 2.11 Transfection

The JetPrime transfection reagent (Polyplus‐Transfection SA, Illkirch, France) was used for transient transfection of cells with expression vectors. Lentiviral particles expressing either control shRNAs (shCon; pLKO.1) or *S100A14* (*SA14*) shRNAs were transduced into HCT/P cells to establish stable cell lines devoid of SA14 expression (Sigma‒Aldrich, St. Louis, MO, USA). The target sequence for silencing *SA14* is as follows: GAGACCCTCATCAAGAACTTT. Three weeks of culture in medium containing 1–2 μg/ml puromycin was used to select cells that had been transduced stably. To produce stable cell lines overexpressing *SA14*, HCT/FuR, HCT/OxaR and CT26 cells were transfected for 48 h with pCMV6 (EV) or pCMV6‐Myc‐DDK‐SA14 vectors using JetPRIME. 3 to 4 weeks were spent selecting transfected cells with 0.5–2 μg/ml G418.

### 2.12 CRISPR/Cas9‐based *CD274* silencing

The guide RNA sequences were selected using the CHOPCHOP database.^39^ The target sequences for guide RNA are as follows—mouse Cd274: 5′‐GTATGGCAGCAACGTCACGA‐3′; human CD274: 5′‐TACCGCTGCATGATCAGCTA‐3′. Guide RNA sequences were cloned into the pCAG‐SpCas9‐GFP‐U6‐gRNA vector. Cells were plated into six‐well plates and subsequently transfected with pCAG‐SpCas9‐green fluorescent protein (GFP) (as the negative control), pCAG‐SpCas9‐GFP‐sgCd274, or pCAG‐SpCas9‐GFP‐sgCD274 clones. After a 2‐day transfection, GFP‐positive cells were isolated by flow cytometry. We confirmed the silencing of CD274 expression in the GFP‐positive pools by WB analysis.

### 2.13 Pulldown and immunoprecipitation assays

The immunoprecipitation (IP) analysis and the production and purification of hexahistidine (6xHis, His)‐tagged recombinant S100A14 (His‐SA14) or GST‐tagged STAT3 (GST‐STAT3) proteins were carried out as previously reported.^38^, ^40^, ^41^

### 2.14 Limiting dilution assay

The limiting dilution assay was carried out as stated in a previous report.^37^

### 2.15 Transcription factor reporter array

To identify the signalling pathways activated in chemoresistant cells, HCT/P and chemoresistant (HCT/FuR and HCT/OxaR) cells were analysed by using the Cignal 45‐Pathway Reporter Array (Qiagen, Germantown, MD, USA) according to the manufacturer's instructions.

### 2.16 Evaluation of CD8^+^ T‐cell tumour cell killing activity

A CD8^+^ T‐cell isolation kit was used to separate splenic CD8^+^ T cells from freshly isolated spleens of 8‐week‐old male Balb/c mice (Miltenyl Biotec). Isolated CD8^+^ T cells were activated by culturing in RPM 1640 medium (containing 10% FBS, antibiotics and 50 μM β‐mercaptoethanol) for 3 days with anti‐CD3 and anti‐CD28 Abs. CT26‐EV and CT26‐SA14 cells (1 × 10^4^ cells/well) were plated in 96‐well plates and incubated for 1 day. Following incubation, the cells were cocultured for 12 h with activated T cells [at 2:1 (T cells:tumour cells) ratio]. As detailed below, the crystal violet test was used to evaluate cell viability. When necessary, cultured cancer cells were pretreated for 12 h with FU/Oxa (1 μM 5‐FU and 2 μM Oxa in combination) and anti‐PD‐L1 Abs (10 μg), either alone or in combination, before being cocultured with activated T cells [at 2:1 (T cells:tumour cells) ratio]. The crystal violet test was used to determine the viability of tumour cells after they had been fixed in 100% methanol for 30 min at room temperature. Thereafter, fixed cells were stained for 1 h at room temperature with a 0.02% crystal violet solution before being rinsed several times with deionised water. At 570 nm, the absorbance of each well was measured after stained cells were dissolved in a 10% acetic acid solution.

### 2.17 Toxicity test

Serum was taken from mice after cardiac puncture as stated in a previous report^37^ to detect hepatic or renal toxicities following treatment with either chemotherapy (a combination of 5‐FU and Oxa) or anti‐PD‐L1 immunotherapy. Measurements of serum levels of aspartate aminotransferase (AST), alanine aminotransferase (ALT), creatinine and blood urea nitrogen (BUN) were made using a Fuji DRI‐Chem 3500s veterinary hematology analyser (Fuji, Tokyo, Japan).

### 2.18 Cytokine/chemokine array

The Proteome Profiler Mouse Cytokine Array Kit (R&D Systems, Minneapolis, MN, USA) was used to measure the amounts of cytokines and chemokines in the mouse serum, as directed by the manufacturer.

### 2.19 Patients and specimens

All investigations using patient‐derived tissues were performed in accordance with protocols approved by the institutional review boards of Asan Medical Center and Shanghai Cancer Institute. Ninety‐four surgically resected colorectal tumours were collected from 22 institutes in Korea for IHC examination of the SA14 level in CRC tissues. Areas of benign epithelium, dysplasia inside polyps and colon cancer were found in each of the haematoxylin and eosin‐stained sections. Reviewing the patients' medical records yielded the following information: each patient's age, the presence of additional tumours and the outcomes of the most recent follow‐up examination. The pathological features that were analysed were growth patterns, tumour location, tumour size, histological subtype, differentiation status, depth of invasion, lymph node metastases and the existence of lymphatic invasion. In addition, tumours from CRC patients (*n* = 18) at Shanghai Cancer Institute were utilised and evaluated for IHC examination of SA14 and STAT3 levels in normal (paracancerous) and CRC tumour tissues.

### 2.20 Tissue microarray

From morphologically typical regions of formalin‐fixed, paraffin‐embedded blocks, 2‐mm‐diameter tissue cores were extracted. Selected were regions where tumour cells occupied greater than 75% of cells with substantial histological differentiation and were not accompanied by tumour necrosis. The tissue array blocks had two or three cores from each tumour tissue and one core from the associated lymph node metastasis.

### 2.21 Statistics

The data are shown as means ± standard deviations. All in vitro studies were carried out at least three times independently, and a representative result is provided. GraphPad Prism (version 9, GraphPad Software Inc., La Jolla, CA, USA) was used to compute and analyse the data. The two‐tailed Student's *t*‐test, Mann‒Whitney test and one‐way analysis of variance were used to establish statistical significance. To confirm that two test groups had the same variance, an *F*‐test for equality of variances was conducted. To ensure that more than three experimental groups had the same variance, the Brown–Forsythe test was used. The Shapiro‒Wilk test was used to examine whether the data from in vitro or in vivo were normally distributed. *p*‐Values less than .05 were deemed statistically significant. The *χ* ^2^ and Fisher exact tests were used to compare any relationships between immunohistochemical markers and clinicopathological parameters.

## 3 RESULTS

### 3.1 PD‐L1 induces CSC properties in CRC cells, causing chemoresistance

**FIGURE 1:**
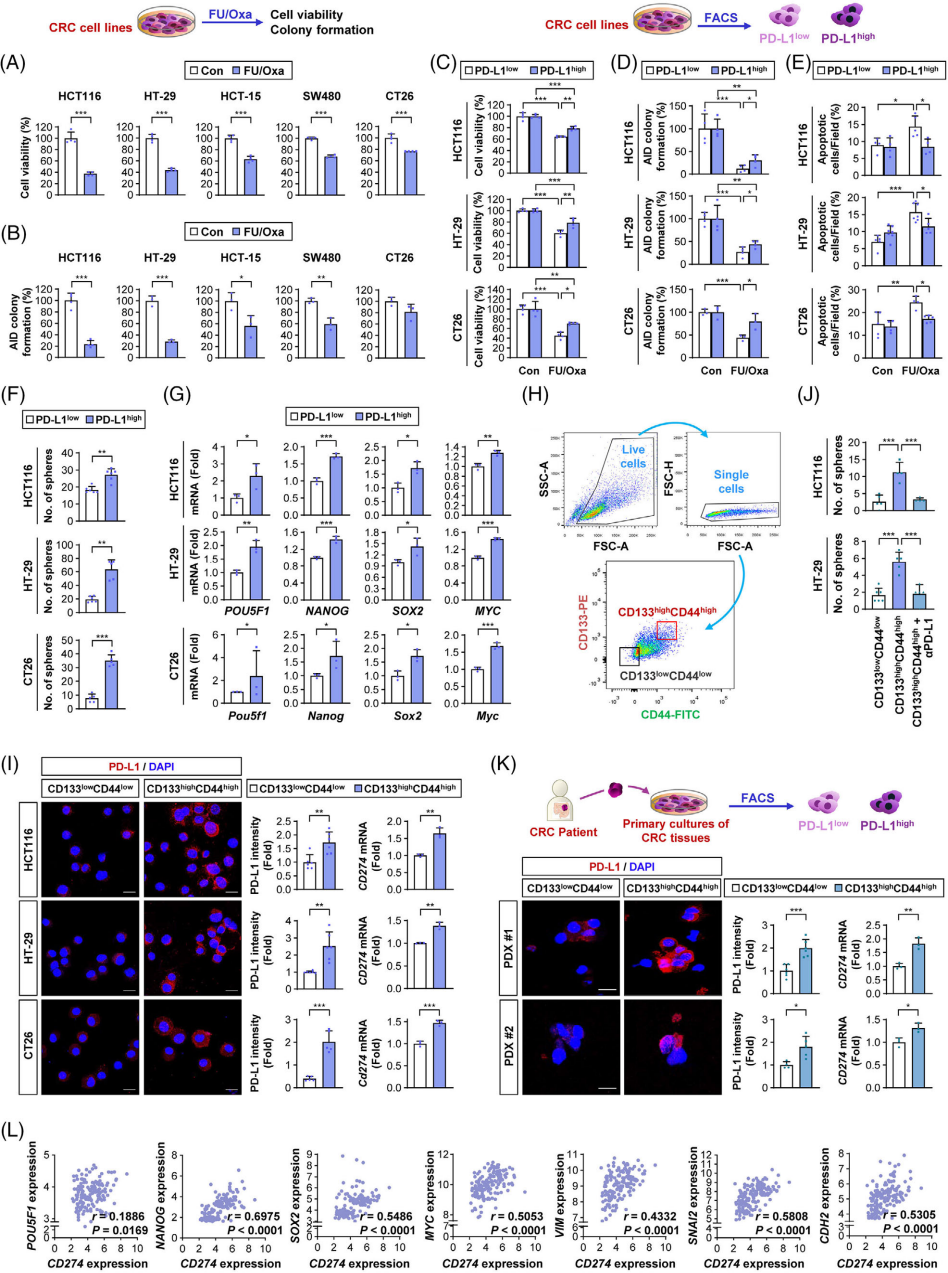
Programmed death‐ligand 1 (PD‐L1) is associated with acquiring cancer stem cell (CSC) properties and chemoresistance in colorectal carcinoma (CRC) cells. (A and B) 3‐[4,5‐Dimethylthiazol‐2‐yl]‐2,5 diphenyl tetrazolium bromide (MTT) (A) and anchorage‐independent (AID) colony formation (B) assays showing the effects of combination treatment with 5‐fluorouracil (5‐FU, 0.2 μM) and oxaliplatin (Oxa, 4 μM) on the viability (A) and colony formation under AID culture conditions (B) of CRC cell lines. (C–G) Effects of a 5‐FU/Oxa combination on cell viability (C), AID colony formation (D) and apoptosis (E) and changes in sphere formation (F) and the expression of putative CSC‐associated markers (G) in intrinsic PD‐L1^high^ CRC subpopulations by comparison with those in PD‐L1^low^ CRC subpopulations determined by MTT assay (C), AID colony formation assay (D), Hoechst 33342 staining for determining chromatin condensation (E) sphere formation assay (F), and real‐time PCR (G). (H) Gating strategy for the isolation of putative CSC and non‐CSC populations in CRC cell lines and primary CRC cells derived from patient‐derived xenograft (PDX) tumours. (I and K) Changes in the level of PD‐L1 expression in CD133^high^CD44^high^ putative CSC subpopulations of the cell line (I) and primary CRC cells derived from PDX tumours (K) by comparison with those in CD133^low^CD44^low^ CRC subpopulations determined by immunofluorescence analysis and real‐time PCR. Scale bars: 20 μm. (J) Regulation of sphere formation ability of the CD133^high^CD44^high^ subpopulation of HCT116 and HT‐29 cells by treatment with anti‐PD‐L1 antibody (10 μg/ml). (L) Analysis of a gene expression omnibus (GEO) dataset (GSE24551) to determine the correlation between PD‐L1 and CSC‐ or epithelial‐mesenchymal transition (EMT)‐associated markers. The significance of the correlation was determined by the Spearman rank correlation test (*n* = 160). The bars represent the mean ± standard deviation (SD); *p < .05, **p < .01 and ***p < .001, as determined by a two‐tailed Student's *t*‐test by comparison with the indicated control (Con) (A, B, F, G, I, K) or one‐way analysis of variance (ANOVA) with Tukey's post hoc test (C–E, J)

We explored the involvement of PD‐L1 in acquiring CSC‐like properties and chemoresistance, especially resistance to FOLFOX‐based chemotherapy, in CRC cells. First, the level of PD‐L1 expression in a selection of CRC cell lines (HCT116, HT‐29, HCT‐15 and SW480) was assessed by flow cytometry and WB analyses. Except for SW480 cells, the PD‐L1 levels on the cell membrane were not much different in HCT116, HT‐29 and HCT‐15 cells (Figure S1A). However, WB analysis showed that HCT116 and HT‐29 cells exhibited markedly reduced PD‐L1 levels compared to HCT‐15 and SW480 cells (Figure S1B). These findings implied broad distributions of PD‐L1 in the membrane, cytoplasm and nucleus, as previously reported.^42^ We then chose human HCT116 and HT‐29 as PD‐L1^low^ expressors and HCT‐15 and SW480 as PD‐L1^high^ expressors. We investigated the relationship between PD‐L1 expression and the response to combinatorial treatment with 5‐FU and Oxa (FU/Oxa). We also included a murine colon cancer cell line (CT26) harboring a prominent level of PD‐L1 expression.^43^, ^44^ In vitro responsiveness to FU/Oxa, measured by 3‐[4,5‐dimethylthiazol‐2‐yl]‐2,5 diphenyl tetrazolium bromide (Figure 1A) and AID colony formation (Figure 1B) assays, revealed that PD‐L1^low^ expressors (HCT116 and HT‐29) exhibited relatively greater FU/Oxa sensitivity than PD‐L1^high^ expressors (HCT‐15, SW480 and CT26).

We hypothesised that a rare chemoresistant PD‐L1^high^ subpopulation may exist in CRC cells and that repeated exposure to chemotherapy may enrich the PD‐L1^high^ subpopulation, causing acquired chemoresistance. Hence, we next analysed FACS‐sorted PD‐L1^high^ and PD‐L1^low^ subpopulations from CRC cells (Figure S1C). As expected, the PD‐L1^high^ subpopulation of CT26 cells showed significantly greater resistance to T‐cell‐mediated cytotoxicity than the corresponding PD‐L1^low^ subpopulation (Figure S1D). Notably, the PD‐L1^low^ subpopulation in SW480 cells (an intrinsic PD‐L1^high^ expressor) showed markedly greater mRNA expression of *CD274*, which encodes the PD‐L1 protein, than did the PD‐L1^high^ subpopulations in HCT116 cells (an intrinsic PD‐L1^low^ expressor) (Figure S1E). Moreover, the PD‐L1^low^ and PD‐L1^high^ subpopulations in SW480 cells showed similar responsiveness to FU/Oxa treatment and sphere‐forming ability (Figure S1F). Hence, we assessed PD‐L1^low^ and PD‐L1^high^ subpopulations in the two PD‐L1^low^ expressors (HCT116 and HT‐29) for CSC‐like properties. The PD‐L1^high^ subpopulations showed greater resistance to 5‐FU/Oxa‐mediated effects on cell viability (Figure 1C) and AID colony formation (Figure 1D) and induction of apoptosis (Figure 1E) compared to their corresponding PD‐L1^low^ populations. These PD‐L1^high^ subpopulations also showed significantly greater sphere‐forming capacity (Figure 1F) and expression of CSC marker genes, including *POU5F1* (which encodes Oct4), *NANOG*, *SOX2*, and *MYC* ^45^, ^46^ (Figure 1G), than did their corresponding PD‐L1^low^ subpopulations. Because IFN‐γ stimulation induces functional PD‐L1 in tumour cells,^47^ we further analysed FACS‐sorted PD‐L1^low^ and PD‐L1^high^ populations after stimulation with IFN‐γ and found consistent results that PD‐L1^high^ subpopulations in HCT116, HT‐29 and CT26 cells exhibited significantly reduced sensitivity to 5‐FU/Oxa treatment and greater capacities for sphere formation compared with PD‐L1^high^ subpopulations in their corresponding parental cells (Figure S1G,H).

We next analysed PD‐L1 levels in a putative CSC subpopulation within CRC cells by adapting the use of Abs against the well‐established CSC markers CD133 and CD44 (Figure 1H).^45^ As shown by immunofluorescence (Figure 1I, left) and real‐time PCR (Figure 1I, right) analyses, the CD133^high^CD44^high^ subpopulation within the three CRC cell lines exhibited significantly greater expression of PD‐L1 protein and *CD274* (which encodes PD‐L1) mRNA levels compared with their corresponding CD133^low^CD44^low^ population. Moreover, treatment of the CD133^high^CD44^high^ subpopulation with a neutralising Ab against PD‐L1 significantly attenuated the sphere‐forming capacity (Figure 1J). We then analysed the SW480 subline, in which PD‐L1 expression was silenced using the CRISPR/Cas9 system. We discovered that knocking down PD‐L1 resulted in downregulated expression of CSC markers, decreased sphere formation and markedly increased apoptotic activities in response to 5‐FU/Oxa treatment, implying the involvement of PD‐L1 in chemoresistance and CSC properties in CRC cells (Figure S2A–D).

To investigate the clinical significance of these findings, we examined PD‐L1 expression in CRC CSCs within patient‐derived tumours. The CD133^high^CD44^high^ subpopulation within two different patient‐derived CRC tissues showed significantly greater expression of PD‐L1 protein and *CD274* mRNA levels than the control population (Figure 1K). Analyses of a publicly available gene expression omnibus (GEO) dataset (GSE24551) from CRC patients indicated positive relationships between *CD274* expression and CSC‐related genes, including *POU5F1*, *NANOG*, *SOX2* and *MYC* (Figure 1L). In line with the association of EMT with acquiring CSC‐like phenotypes,^48^ positive correlations between *CD274* expression and EMT‐related genes, including *VIM* (which encodes vimentin), *SNAI2* (which encodes Slug) and *CDH2* (which encodes N‐cadherin), were also observed (Figure 1L). Thus, PD‐L1 expression may contribute not only to immune evasive phenotypes but also to the development of CSC‐like characteristics, causing chemoresistance in CRC cells.

### 3.2 CRC sublines carrying resistance to 5‐FU or Oxa display upregulation of PD‐L1 expression and CSC‐like properties

**FIGURE 2:**
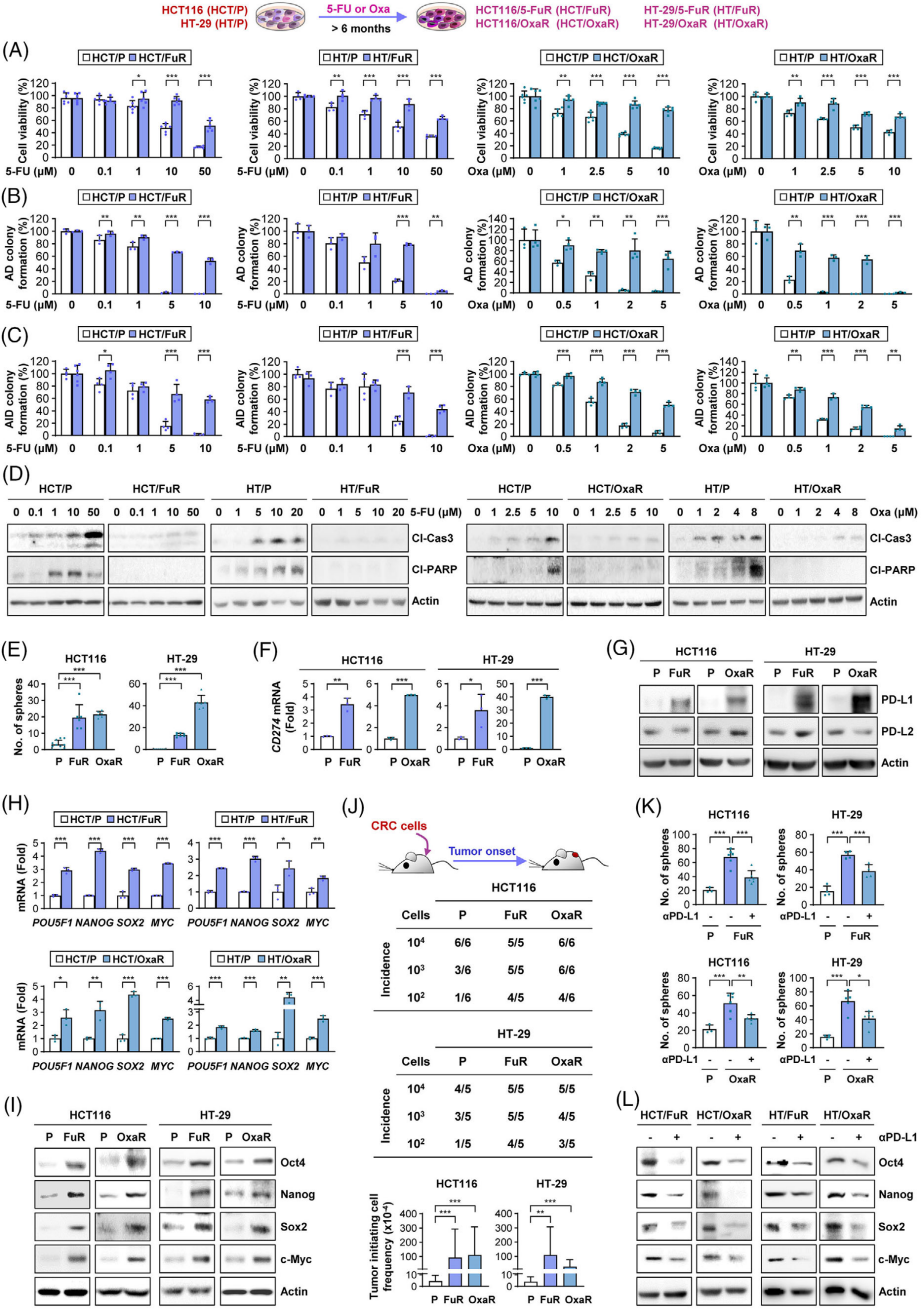
Cancer stem cell (CSC)‐like phenotypes and programmed death‐ligand 1 (PD‐L1) expression are upregulated in chemoresistant colorectal carcinoma (CRC) cells. (A–D) Establishment of chemoresistant CRC sublines. Determination of the reduced effects of chemotherapeutic agents [5‐fluorouracil (5‐FU) or oxaliplatin (Oxa)] on the inhibition of cell viability and colony formation and induction of apoptosis in chemoresistant CRC sublines compared with those in corresponding parental cells by 3‐[4,5‐dimethylthiazol‐2‐yl]‐2,5 diphenyl tetrazolium bromide (MTT) assay (A), anchorage‐dependent (AD) colony formation assay (B), anchorage‐independent (AID) colony formation assay (C) and Western blot analysis (D). (E and J) Evaluation of increases in the CSC population in chemoresistant CRC cells by sphere formation assay (E) and limiting dilution assay (J). Extreme limiting dilution analysis (ELDA) evaluation of the tumour‐initiating cell frequency (J). (F–I) Determination of the regulation of the mRNA and protein expression of PD‐L1 (F and G) and CSC‐associated markers (H and I) by real‐time PCR (F and H) and Western blot analysis (G and I). (K and L) Evaluation of the modulation of sphere formation (K) and the expression of stemness markers (L) by blockade of PD‐L1 with the PD‐L1 neutralising antibody by sphere formation assay (K) and Western blot analysis (L). The bars represent the mean ± standard deviation (SD); *p < .05, **p < .01 and ***p < .001, as determined by a two‐tailed Student's *t*‐test by comparison with the indicated control (A–C, F, H) or one‐way analysis of variance (ANOVA) with Dunnett's post hoc test (E, J, K). Cl‐Cas3: cleaved caspase‐3; Cl‐PARP: cleaved poly‐(ADP‐ribose) polymerase; HCT: HCT116; HT: HT‐29

To obtain PD‐L1^high^ CRC cells with chemoresistance, HCT116 and HT‐29 cells were repeatedly exposed to 5‐FU or Oxa for more than 6 months. Compared to their parental HCT116 and HT‐29 cells (HCT/P and HT/P, hereafter), the surviving sublines after prolonged exposure to 5‐FU (HCT/FuR and HT/FuR, hereafter) or Oxa (HCT/OxaR and HT/OxaR, hereafter) demonstrated greatly increased viability (Figure 2A) and AD (Figure 2B) and AID (Figure 2C) colony‐forming capacities and reduced apoptotic activities (Figure 2D) in response to 5‐FU or Oxa. Importantly, these chemoresistant sublines consistently exhibited upregulation of sphere‐forming capacities (Figure 2E) and *CD274* expression (Figure 2F) and PD‐L1 expression without overt changes in PD‐L2 expression (Figure 2G) in comparison with their parental cells. Increases in mRNA (Figure 2H) and protein (Figure 2I) expression of CSC‐regulating pluripotent transcription factors, including Oct4, Nanog, Sox2 and Myc,^46^ were also observed in these chemoresistant sublines compared with their parental cells. The in vivo limiting dilution assay confirmed the greater tumourigenic potential of these chemoresistant sublines compared with their parental cells (Figure 2J). Importantly, treatment of chemoresistant CRC sublines with a neutralising Ab against PD‐L1 significantly attenuated sphere‐forming capacity (Figure 2K) and CSC marker expression (Figure 2L). We then analysed HCT/FuR cells that had PD‐L1 expression knocked down utilising the CRISPR/Cas9 system. We found that silencing CD274 expression resulted in decreases in the expression of CSC markers, sphere‐forming ability and colony formation in the presence of 5‐FU and sensitised cells to 5‐FU‐induced apoptosis, implying the involvement of PD‐L1 in chemoresistance and CSC properties in CRC cells (Figure S2E–H). These results indicate that PD‐L1 expression granted chemoresistant CRC sublines CSC phenotypes, contributing to tumour development.

### 3.3 Loss of SA14 is implicated in chemoresistance and development of CSC‐like features in PD‐L1^high^ CRC cells and worse clinical outcomes in CRC patients

**FIGURE 3:**
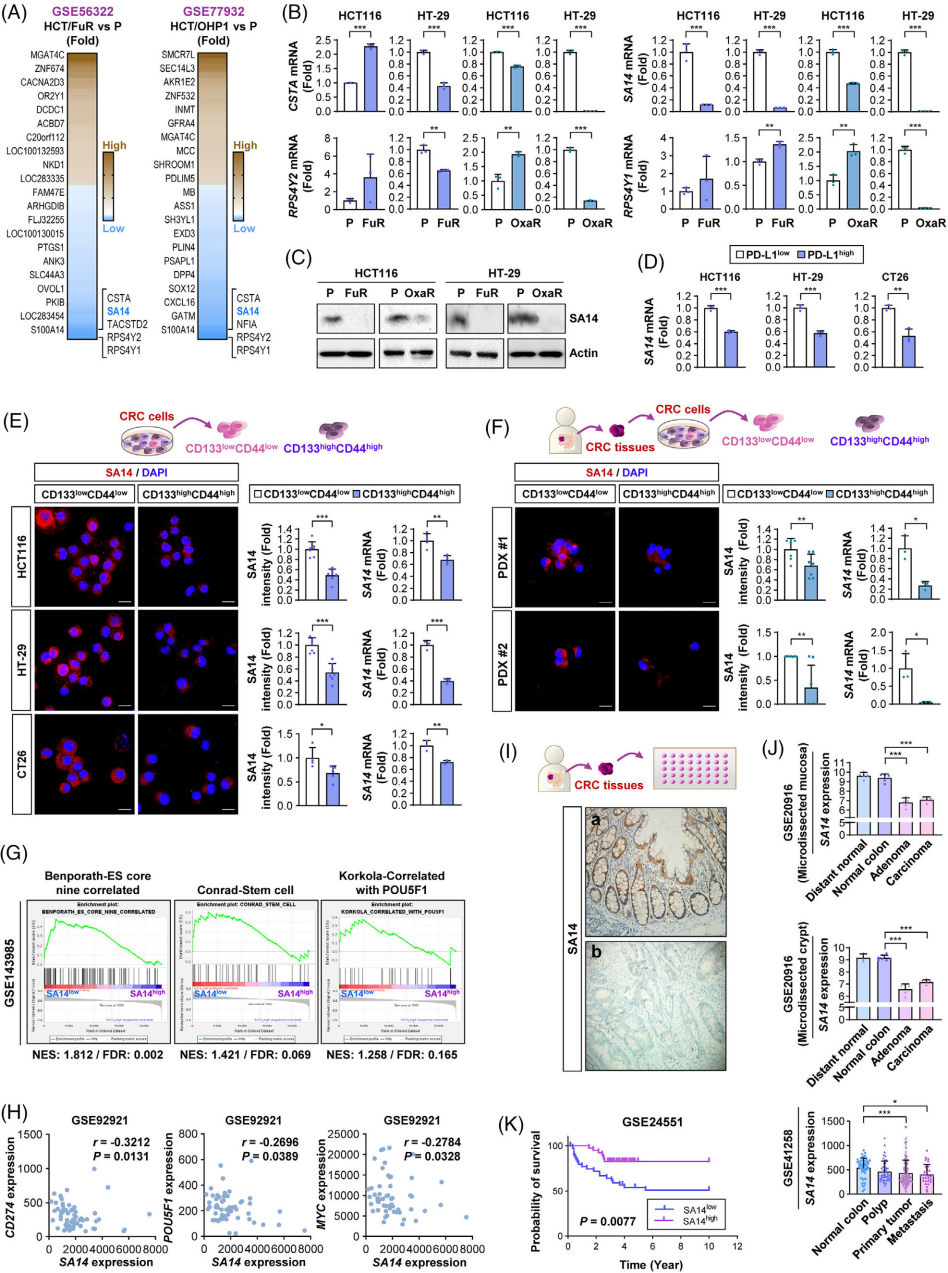
Reduction in the S100A14 (SA14) expression level is correlated with the expression of stemness markers and programmed death‐ligand 1 (PD‐L1) in colorectal carcinoma (CRC) cells and poor clinical outcomes in patients with CRC. (A) Heatmaps showing differentially expressed genes in the chemoresistant populations compared with those in corresponding parental cell populations in two publicly available datasets. (B) Real‐time PCR analysis for the modulation of the expression of commonly downregulated genes in two analysed datasets [*S100A14* (*SA14*), *CSTA*, *RPS4Y1* and *RPS4Y2*] in the established chemoresistant sublines. (C) Determination of the regulation of the protein of SA14 in the established chemoresistant sublines compared with those in the corresponding parental cells by Western blot analysis. (D) Determination of the regulation of the mRNA expression of SA14 in the PD‐L1^high^ population of CRC cells compared with that in the PD‐L1^low^ population of CRC cells by real‐time PCR. (E and F) Immunofluorescence staining and real‐time PCR analysis for the modulation of SA14 levels in the putative cancer stem cell (CSC) population (CD133^high^CD44^high^) of CRC cell lines (E) and in the putative CSC population (CD133^high^CD44^high^) of two patient‐derived xenograft (PDX)‐derived primary CRC cells (F) compared with those in the corresponding non‐CSC population (CD133^low^CD44^low^). Scale bars: 20 μm. (G) Gene set enrichment analysis (GSEA) of a GSE143985 dataset showing the enrichment of CSC‐associated gene sets in the SA14^low^ populations compared with those in the SA14^high^ populations. (H) Spearman correlation coefficient showing the relationship between *SA14* expression and the expression of PD‐L1 and stemness markers (*POU5F1* and *MYC*) in tumours derived from patients with CRC, determined by analysis of a GSE92921 dataset (*n* = 59). (I) Representative images of SA14 expression in normal colonic mucosa and cancers. (a) A representative image showing normal colonic surface epithelia show strong SA14 expression (20× magnification); (b) a representative image showing a case with SA14 expression loss (20× magnification). (J and K) Analysis of gene expression omnibus (GEO) datasets to determine the regulation of SA14 expression in CRC tumours by comparison with normal CRC tissues (J), metastasised tumours by comparison with primary tumours (J), and survival of patients with CRC (K). The bars represent the mean ± standard deviation (SD); *p < .05, **p < .01 and ***p < .001, as determined by a two‐tailed Student's *t*‐test (B, D, E, F), Mann‒Whitney test (F), one‐way analysis of variance (ANOVA) with Dunnett's post hoc test (J, top and middle), or Kruskal‒Wallis test with Dunn's post hoc test (J, bottom). HCT: HCT116; HT: HT‐29

To examine the mechanism governing the control of PD‐L1 expression, CSC‐associated phenotypes and chemoresistance in CRC cells, we analysed publicly available datasets for the gene expression profiles of HCT116 sublines that showed resistance to 5‐FU (GSE56322) or Oxa (GSE77932). Among a number of genes that were either upregulated (fold >2) or downregulated (fold <0.5), four genes (*SA14*, *CSTA*, *RPSAY1* and *RPSAY2*) were commonly downregulated in the two chemoresistant HCT116 subpopulations (Figure 3A). Among these genes, *SA14* was consistently downregulated in the four chemoresistant sublines established in our study in comparison to their parental cells (Figure 3B). We validated the downregulation of SA14 protein in the chemoresistant sublines in comparison to that in their parental cells (Figure 3C). We determined whether other chemotherapeutic agents may also regulate SA14 expression. Analysis of a publicly available dataset revealed downregulation of SA14 expression in irinotecan‐resistant LoVo cells (Figure S3A). We further analysed SA14 expression in HT‐29 and HCT116 cells that survived treatment with trifluridine for 7 days and found consistently reduced SA14 expression in the surviving subpopulations (Figure S3B). These findings suggest chemotherapy‐mediated SA14 downregulation in CRC cells.

We confirmed markedly reduced *SA14* expression levels in PD‐L1^high^ subpopulations within HCT116, HT‐29 and CT26 cells compared with their corresponding PD‐L1^low^ control subpopulations (Figure 3D). CD133^high^CD44^high^ potential CSC subpopulations within HCT116, HT‐29 and CT26 cells (Figure 3E) and two CRC patient tumours (Figure 3F) also showed reduced levels of SA14 expression compared with their corresponding control subpopulations. Gene set enrichment analysis using a GEO dataset (GSE143985) found that gene sets linked with stem cells were significantly enriched in SA14^low^ populations, with a false discovery rate of less than 0.25 (Figure 3G). Inverse correlations between *SA14* and *CD274*, *POU5F1*, or *MYC* mRNA expression were also confirmed by scrutinising a publicly available dataset GSE92921 (Figure 3H).

We then performed IHC analysis of SA14 expression in CRC patient tissues (*n* = 94). SA14 expression was prominent on the cellular membrane of colon cells (Figure 3I‐a). The intensity of the staining was moderate to intense, and it was uniformly distributed. Table S4 summarises the associations between SA14 expression and clinicopathological characteristics. We have correlated the loss (Figure 3I‐b) of SA14 expression level with several clinicopathological characteristics. Among 94 patients with colon cancer, loss of SA14 expression was seen more often in distal (jejunum or ileum) malignancies (43 of 66, 65.2%) than in proximal (duodenum) tumours (*p* = .038). Cases with lymphovascular invasion (26 of 34, 76.5%) had a greater decrease in SA14 expression than those without lymphovascular invasion (29 of 60, 48.3%; *p* = .007). In addition, patients with lymph node metastasis (36 of 52, 69.2%) or distant metastasis (31 of 42, 73.8%) had a greater loss of SA14 expression than those without lymph node metastasis (19 of 42, 45.2%; *p* = .016) or distant metastasis (24 of 52, 46.2%; *p* = .006), respectively. There was no significant relationship between SA14 expression loss and other clinicopathological variables, such as age, tumour size, differentiation, depth of invasion or perineural invasion status. Analysis of two independent GEO datasets also revealed that *SA14* mRNA was mostly downregulated in tissue from CRC patients compared with their counterparts with normal tissue (Figure 3J). Next, we evaluated how SA14 expression affected the clinical outcomes of CRC patients. Survival analysis using a GEO dataset indicated that patients with CRC with low SA14 expression displayed significantly decreased overall survival rates (Figure 3K). Therefore, in CRC cells, SA14 expression appeared to have an inverse correlation with PD‐L1 expression, particularly in those with CSC phenotypes, and acted as a poor prognostic marker in CRC patients.

### 3.4 SA14 regulates PD‐L1 expression, chemoresistance and CSC phenotypes in CRC cells

**FIGURE 4:**
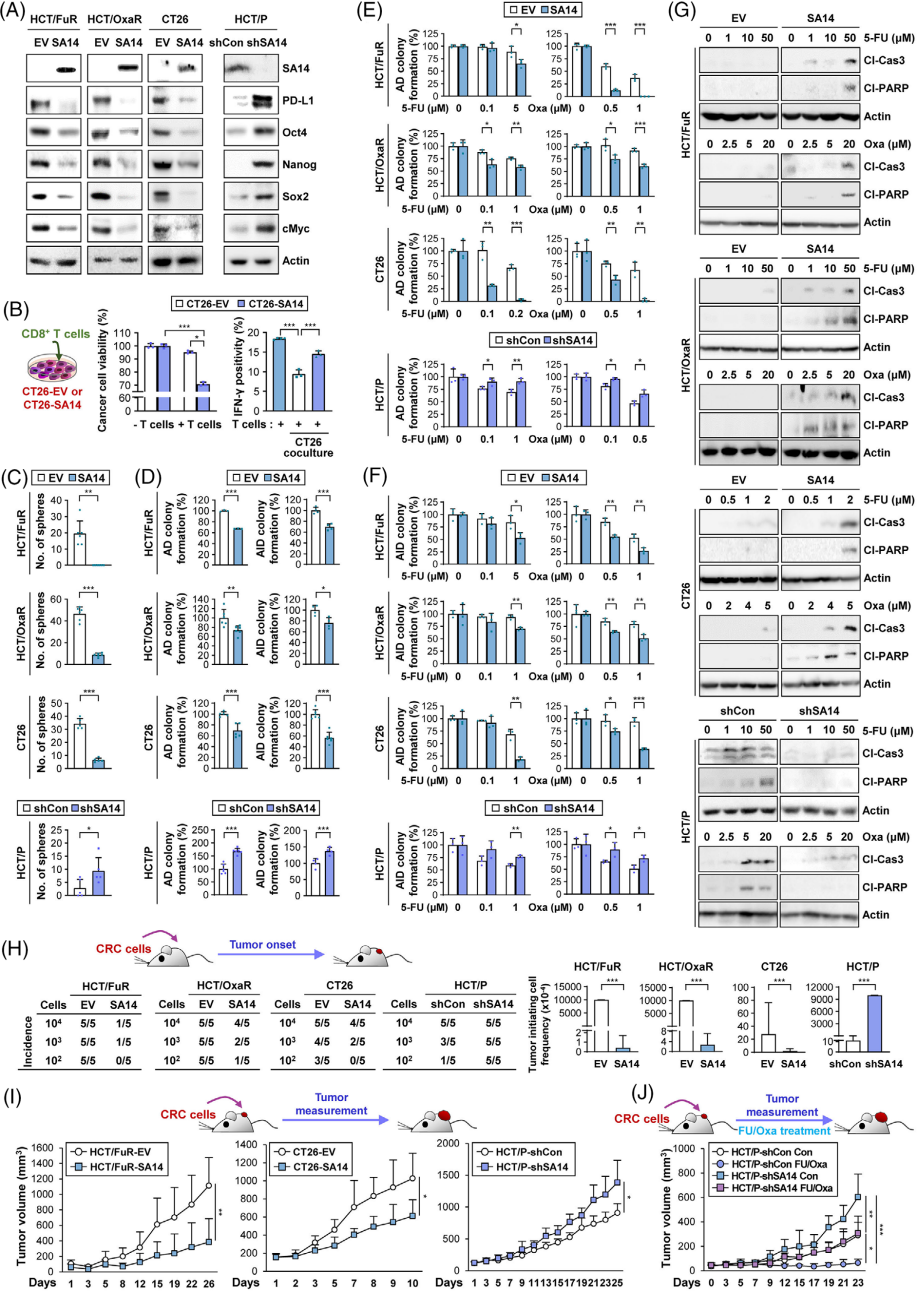
SA14 downregulation is associated with the acquisition of cancer stem cells (CSC) phenotypes, programmed death‐ligand 1 (PD‐L1) expression, and chemoresistance in colorectal carcinoma (CRC) cells. (A, C–J) Regulation of the expression of stemness markers and PD‐L1 (A), sphere formation (C), anchorage‐dependent (AD) colony formation in the absence (D, left) or presence of 5‐fluorouracil (5‐FU) or oxaliplatin (Oxa) (E), anchorage‐independent (AID) colony formation in the absence (D, right) or presence of 5‐FU or Oxa (F), chemotherapy‐induced apoptosis (G), tumourigenicity (H), tumour growth (HCT/FuR‐EV and HCT/FuR‐SA14: *n* = 5; CT26‐EV and CT26‐SA14: *n* = 6; HCT/P‐shCon and HCT/P‐shSA14: *n* = 5) (I), and in vivo chemosensitivity (HCT/P‐shCon, Con: *n* = 6; FU/Oxa: *n* = 6; HCT/P‐shSA14, Con: *n* = 5; FU/Oxa: *n* = 5) (J) by overexpression of SA14 in chemoresistant CRC cells or knockdown of SA14 expression in parental CRC cells determined through Western blot analysis (A and G), sphere formation assay (C), AD colony formation assay (D and E), soft agar colony formation assay (D and F), limiting dilution assay (H), extreme limiting dilution analysis (ELDA) evaluation for the tumour‐initiating cell frequency (H), and tumour xenograft experiment (I and J). (B) Left: Crystal violet assay showing the regulation of the tumour cell killing activity of T cells by SA14 overexpression in CT26 cells. Right: Flow cytometric analysis showing changes in the cancer cell‐mediated regulation of T‐cell activity, as indicated by interferon‐gamma (IFN‐γ) positivity, by SA14 overexpression in CT26 cells. The bars represent the mean ± standard deviation (SD); *p < .05, **p < .01 and ***p < .001, as determined by a two‐tailed Student's *t*‐test (C–F, I), Mann‒Whitney test (C), or one‐way analysis of variance (ANOVA) with Tukey's post hoc test (B and J). Cl‐Cas3: cleaved caspase‐3; Cl‐PARP: cleaved poly‐(ADP‐ribose) polymerase; Con: control; EV: empty vector; HCT: HCT116; HT: HT‐29

To obtain clear evidence supporting the involvement of SA14 in PD‐L1 expression and its function in regulating immune evasion and CSC phenotypes, we established HCT/FuR, HCT/OxaR and CT26 cells, in which SA14 overexpression was stably enforced by transfection (HCT/FuR‐SA14, HCT/OxaR‐SA14 and CT26‐SA14 cells), and HCT/P cells, in which shRNA‐mediated silencing of SA14 expression was induced (HCT/P‐shSA14) (Figure 4A). We confirmed that CT26‐SA14 cells were more sensitive to CD8^+^ T‐cell‐mediated cytotoxicity than CT26‐EV cells (Figure 4B, left). We further determined the level of IFN‐γ positivity, a marker for activated T cells,^42^ in CD8^+^ T cells cocultured with either CT26‐EV or CT26‐SA14 cells. Flow cytometric analysis revealed that CD8^+^ T cells cocultured with CT26‐SA14 cells displayed increased IFN‐γ positivity compared with those cocultured with CT26‐EV cells (Figure 4B, right). The established cell lines with enforced overexpression of SA14 revealed obvious decreases in protein (Figure 4A) and mRNA (Figure S3C) expression levels of PD‐L1 and CSC‐associated markers and reduced capacities for sphere formation (Figure 4C, top) compared with their corresponding control cells transfected with EV. Conversely, the established cell line with downregulation of SA14 revealed obviously increased expression of PD‐L1 and CSC‐associated markers (Figures 4A and S3C, bottom) and an enhanced capacity for sphere formation (Figure 4C, bottom) in comparison with the corresponding parental cells transfected with shCon. We additionally assessed the effects of SA14 on the expression level of a subset of immune checkpoint‐related genes, including *CD80*, *CD86*, *CD276*, *LGALS3*, *CD112* and *CD155*.^49^ The level of these genes expression was inconsistently changed by SA14 modulation (Figure S3D). We further confirmed that HCT/FuR‐SA14, HCT/OxaR‐SA14 and CT26‐SA14 cells had reduced capacities for colony formation under AD and AID culture conditions (Figure 4D) compared to their corresponding control cells. The SA14‐overexpressing cells also showed significantly increased responses to 5‐FU and Oxa treatment, as shown by decreases in AD (Figure 4E) and AID (Figure 4F) colony formation and increases in apoptotic cell death (i.e., caspase‐3 and poly‐(ADP‐ribose) polymerase cleavages) (Figure 4G). Conversely, HCT/P‐shSA14 cells exhibited substantial reversion of the aforementioned phenotypes compared to the control (Figure 4D–G).

We next determined the tumourigenic potential of these generated cell populations. A limiting dilution assay in vivo revealed that the tumourigenic potential of HCT/FuR‐SA14, HCT/OxaR‐SA14 and CT26‐SA14 cells was markedly reduced in comparison to that of their corresponding control cells, while HCT/P‐shSA14 cells displayed a significant increase in tumourigenic potential compared to HCT/P‐shCon cells (Figure 4H). Once developed, HCT/FuR‐SA14 xenograft and CT26‐SA14 allograft tumours grew substantially slower than their control tumours, while HCT/P‐shSA14 xenografts showed significantly faster growth than HCT/P‐shCon xenografts (Figure 4I). Notably, CT26‐SA14 allograft tumours had more tumour‐infiltrating CD8^+^ T cells than CT26‐EV tumours (Figure S3E). Moreover, during treatment with chemotherapy (5‐FU and Oxa in combination, 5‐FU/Oxa), HCT/P‐shSA14 xenograft tumours revealed substantially faster growth than HCT/P‐shCon xenograft tumours (Figure 4J). These data suggest that SA14 regulates immune evasive capacities, CSC properties and chemoresistance in CRC cells by suppressing PD‐L1 expression.

### 3.5 STAT3 expression is elevated in PD‐L1^high^ chemoresistant CRCs and acts as a downstream effector for SA14‐mediated transcriptional regulation of PD‐L1 and CSC markers

**FIGURE 5:**
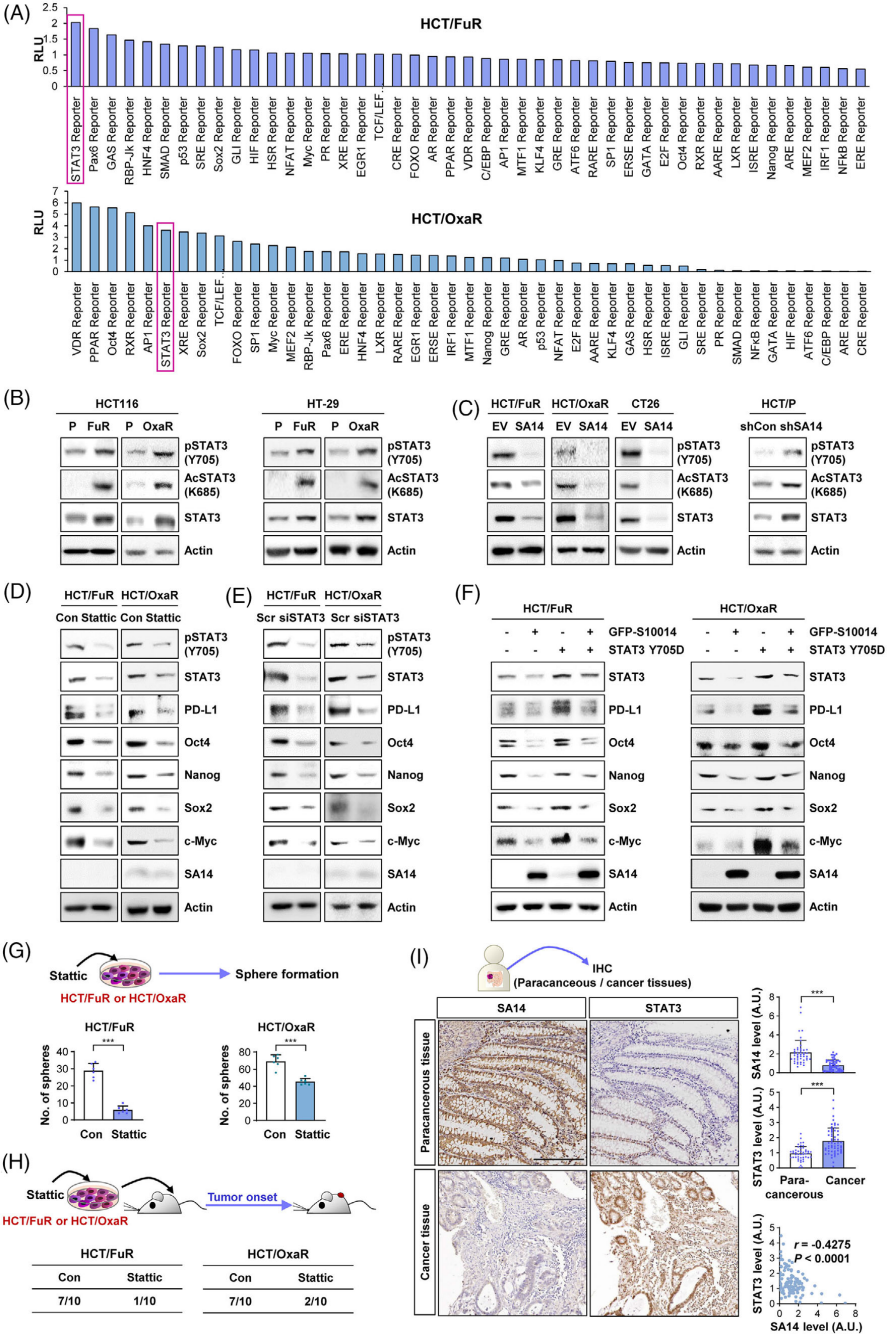
Upregulation of total and phosphorylated signal transducer and activator of transcription 3 (STAT3) levels in chemoresistant colorectal carcinoma (CRC) cells and association of S100A14 (SA14) with the regulation of the expression and activity of STAT3 in CRC cells. (A) Cignal 45‐pathway reporter array analysis for the regulation of the activity of several transcription factors in chemoresistant CRC sublines by comparison with that in parental cells (HCT/P). (B) Determination of the regulation of the expression and phosphorylation of STAT3 in chemoresistant CRC cells by Western blot analysis. (C) Determination of the regulation of the expression and phosphorylation of STAT3 by modulation of SA14 expression in parental and chemoresistant CRC cells by Western blot analysis. (D and E) Modulation of the expression of programmed death‐ligand 1 (PD‐L1) and stemness markers along with total and phosphorylated forms of STAT3 by treatment with Stattic (1 μM, D) or transient transfection with STAT3 siRNAs (E) by Western blot analysis. (F) Modulation of the SA14‐mediated suppression of PD‐L1 and stemness marker expression by overexpression of phosphor‐mimetic mutant form of STAT3 (STAT3 Y705D) by Western blot analysis. (G and H) Regulation of sphere formation ability (G) and tumourigenicity (H) of chemoresistant CRC cells by treatment with Stattic (1 μM) for 1 day by sphere formation assay (G) and tumour xenograft experiment (H). Evaluation of tumourigenicity was performed by inoculation of a small number of cells (50,000 cells/spot) inoculated into the right flanks of NOD/SCID mice. (I) Regulation of SA14 and STAT3 and their Spearman correlation determined by immunohistochemistry analysis of patient‐derived CRC and adjacent normal colon tissues. Scale bar: 200 μm. The bars or dots represent the mean ± standard deviation (SD); *p < .05, **p < .01 and ***p < .001, as determined by a two‐tailed Student's *t*‐test by comparison with the vehicle‐treated control (G and I). Con: control; EV: empty vector; HCT: HCT116; HT: HT‐29

To investigate the mechanisms that are implicated in PD‐L1 expression controlled by SA14 in CRC cells, we screened the activity of several transcription factors in HCT/FuR and HCT/OxaR sublines by using a commercially available transcription factor reporter array. We found that STAT3 reporter activity was consistently upregulated in the two sublines (Figure 5A). Notably, HCT/FuR, HCT/OxaR, HT/FuR and HT/OxaR sublines revealed increases in both total and activated [phosphorylated (Y705) or acetylated (K685)] STAT3 in comparison to their parental cells (Figure 5B). Moreover, these total and activated STAT3 expression levels were markedly reduced in HCT/FuR‐SA14, HCT/OxaR‐SA14 and CT26‐SA14 cells but upregulated in HCT/P‐shSA14 cells in comparison with those in their control cells (Figure 5C).

We next examined whether inactivation of STAT3 would reverse the genotypic and phenotypic changes in the chemoresistant sublines. Upon treatment with the STAT3 inhibitor Stattic^50^ (Figure 5D) or transfection with STAT3 siRNAs (Figure 5E), the expression of PD‐L1 and CSC markers, including Oct4, Nanog, SOX2 and c‐Myc, was markedly reduced, while SA14 expression remained unaffected. In contrast, overexpression of SA14 attenuated constitutively active STAT3 (STAT3 Y705D)‐mediated upregulation of these protein expression (Figure 5F). Moreover, pretreatment with Stattic suppressed the in vitro sphere formation capacity (Figure 5G) and in vivo tumourigenicity (Figure 5H) in HCT/FuR and HCT/OxaR cells. Based on a previous report showing STAT3‐mediated regulation of S100 proteins, including SA14,^51^ we examined possible modulation of SA14 expression by STAT3 activation by utilising HCT116 cells, in which STAT3 was constitutively active by transfection with constitutively active STAT3 (STAT3 Y705D). We found that SA14 expression was minimally affected by constitutive activation of STAT3 (Figure S4A). Hence, the effects of STAT3 on SA14 expression appear to be cell type dependent.

We further validated the correlation between SA14 and STAT3 expression in CRC by performing IHC analysis of tumours from CRC patients (*n* = 18). We observed significantly reduced SA14 and increased STAT3 expression levels in the tumours compared to their corresponding adjacent normal (paracancerous) tissues and a substantial inverse association between the levels of SA14 and STAT3 expression in tissues (Figure 5I). Collectively, these findings imply that SA14 is a crucial regulator of STAT3 expression and CSC phenotypes in CRC cells.

### 3.6 SA14 destabilises STAT3 protein through direct binding and subsequently induces proteasome‐mediated degradation

**FIGURE 6:**
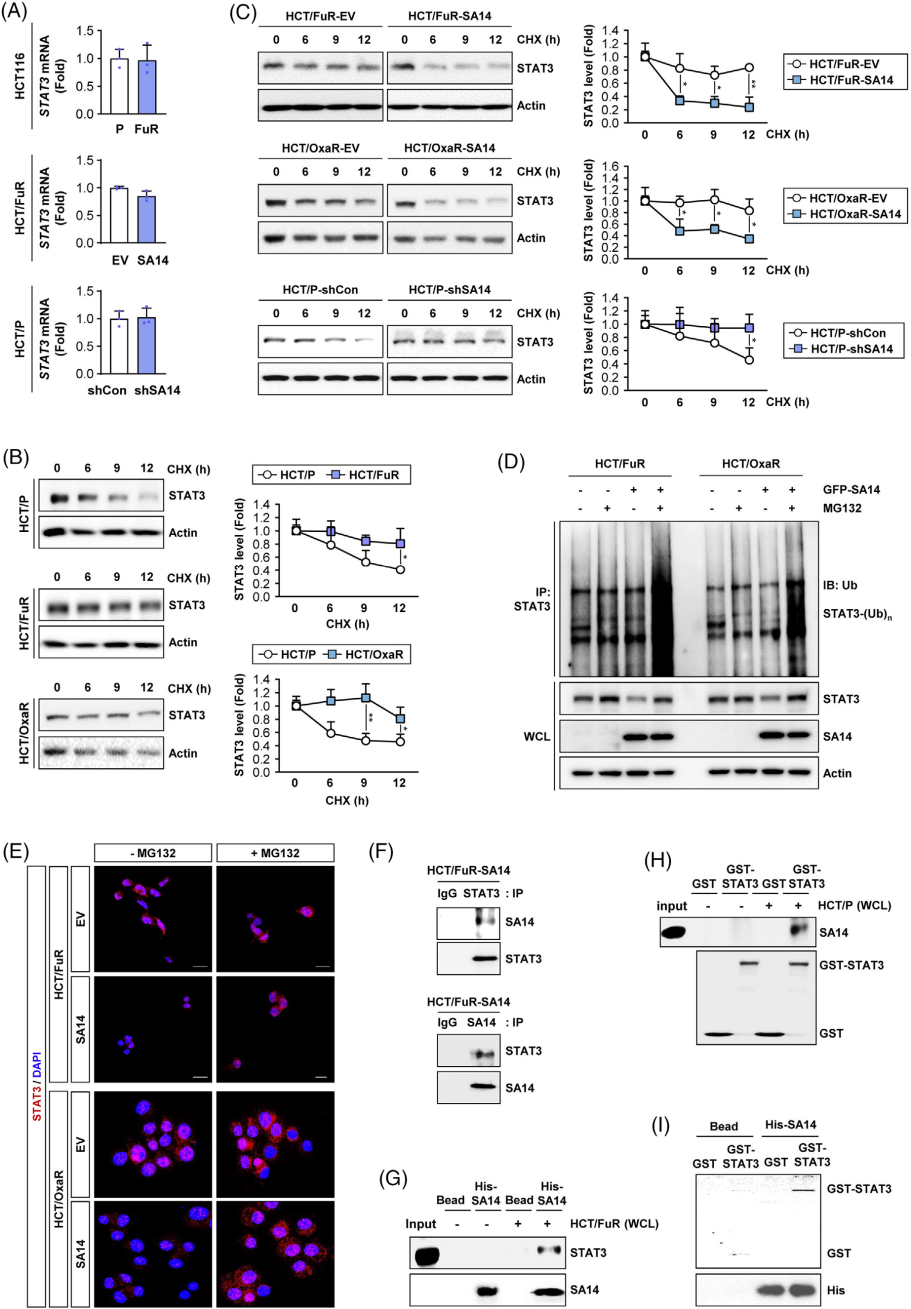
SA14 regulates signal transducer and activator of transcription 3 (STAT3) through direct binding. (A) Real‐time PCR analysis for the determination of regulation of *STAT3* mRNA expression in HCT116 cells (HCT/P), 5‐fluorouracil (5‐FU)‐resistant HCT116 cells (HCT/FuR), and cells carrying modulated SA14 expression (HCT/P‐shSA14 or HCT/FuR‐SA14). (B and C) Changes in the protein stability of STAT3 in parental and chemoresistant HCT116 cells (HCT/P, HCT/FuR and HCT/OxaR cells) (B) and those carrying modulated SA14 expression [HCT116 cells with knockdown of SA14 (HCT/P‐shSA14), 5‐FU‐resistant HCT116 cells with SA14 overexpression (HCT/FuR‐SA14) and oxaliplatin (Oxa)‐resistant HCT116 cells with SA14 overexpression (HCT/OxaR‐SA14)] (C) after treatment with cycloheximide (CHX, 100 μg/ml) for up to 12 h by comparison with corresponding control cells through Western blot analysis. Densitometric analysis was performed using ImageJ software. The dots represent the mean ± standard deviation (SD); *p < .05 and **p < .01, as determined by a two‐tailed Student's *t*‐test by comparison with the vehicle‐treated control (B and C). (D) Transient transfection of HCT/P, HCT/FuR and HCT/OxaR cells with ubiquitin (Ub) or SA14 expression vectors and then treated with MG132 (10 μM) for 6 h. Immunoprecipitation of cell lysates with the anti‐STAT3 antibody, followed by Western blot analysis with the anti‐Ub antibody. Western blot analysis included whole‐cell lysates (WCL). (E) Immunofluorescence analysis showing the regulation of STAT3 by SA14 overexpression in the absence or presence of MG132. Scale bars: 20 μm. (F) Determination of the interaction between STAT3 and SA14 by immunoprecipitation of HCT/FuR‐SA14 cell lysates using anti‐STAT3 or anti‐SA14 antibodies, followed by Western blot analysis for SA14 and STAT3 expression. (G and H) Pull‐down assays to determine the interaction between SA14 and STAT3 by incubating Ni‐NTA agarose‐bound recombinant His‐SA14 (His‐SA14) (G) or glutathione (GSH)‐agarose‐bound recombinant glutathione‐S‐transferase (GST)‐STAT3 (H) proteins with WCL from HCT/FuR cells (G) or HCT/P cells (H). Ni‐NTA agarose (G) or GSH‐agarose‐bound GST (H) was used to ensure specific interactions. (I) Pull‐down assays to determine the direct binding between recombinant SA14 and STAT3 proteins. Ni‐NTA agarose‐bound recombinant His‐SA14 (His‐SA14) and purified GST‐STAT3 proteins were used. Purified GST protein is the control for specific interaction. Con: control; EV: empty vector

We then investigated how SA14 controls STAT3 expression. We observed that *STAT3* transcription did not significantly differ between HCT/FuR versus HCT/P cells, HCT/FuR‐SA14 versus HCT/FuR‐EV and HCT/P‐shSA14 versus HCT/P‐shCon cells (Figure 6A). Notably, HCT/FuR and HCT/OxaR cells showed increases in the half‐life of STAT3 protein compared with HCT/P cells (Figure 6B). Moreover, the half‐life of STAT3 was shorter in HCT/FuR‐SA14 and HCT/OxaR‐SA14 cells but longer in HCT/P‐shSA14 cells than in their controls (Figure 6C). These results imply posttranslational regulation of STAT3 by SA14. Upon a proteasome inhibitor MG132 treatment, STAT3 polyubiquitination after MG132 treatment was markedly increased in HCT/FuR and HCT/OxaR cells, in which SA14 overexpression was enforced (Figure 6D). Moreover, MG132 treatment markedly restored STAT3 expression in HCT/FuR and HCT/OxaR cells, as evidenced by immunoblots of the whole‐cell lysates (Figure 6D). Consistently, immunofluorescence analysis showed that nuclear STAT3 levels were markedly lower in HCT/FuR‐SA14 and HCT/OxaR‐SA14 cells than in HCT/FuR and HCT/OxaR cells, respectively, and were obviously restored by treatment with MG132 (Figure 6E). These results indicated that SA14 destabilises STAT3 protein through proteasome‐mediated degradation.

We then explored the capacity of SA14 to interact with STAT3. Coimmunoprecipitation analysis using STAT3 and SA14 precipitates of MG132‐pretreated HCT/FuR‐SA14 lysates showed a clear interaction between SA14 and STAT3 (Figure 6F). Consistently, pull‐down analysis using bacterial His‐tagged recombinant SA14 protein (His‐SA14) (Figure 6G) and GST‐tagged STAT3 protein (GST‐STAT3) (Figure 6H) demonstrated the ability of SA14 and STAT3. We finally confirmed a direct interaction between SA14 and STAT3 proteins by using His‐SA14 and GST‐STAT3 (Figure 6I). These results collectively suggest that SA14 directly interacts with STAT3 and causes its destabilisation through proteasomal degradation.

### 3.7 SA14 is a possible biomarker for predicting the antitumour efficacy of PD‐L1‐targeted immunotherapy and chemotherapy

**FIGURE 7:**
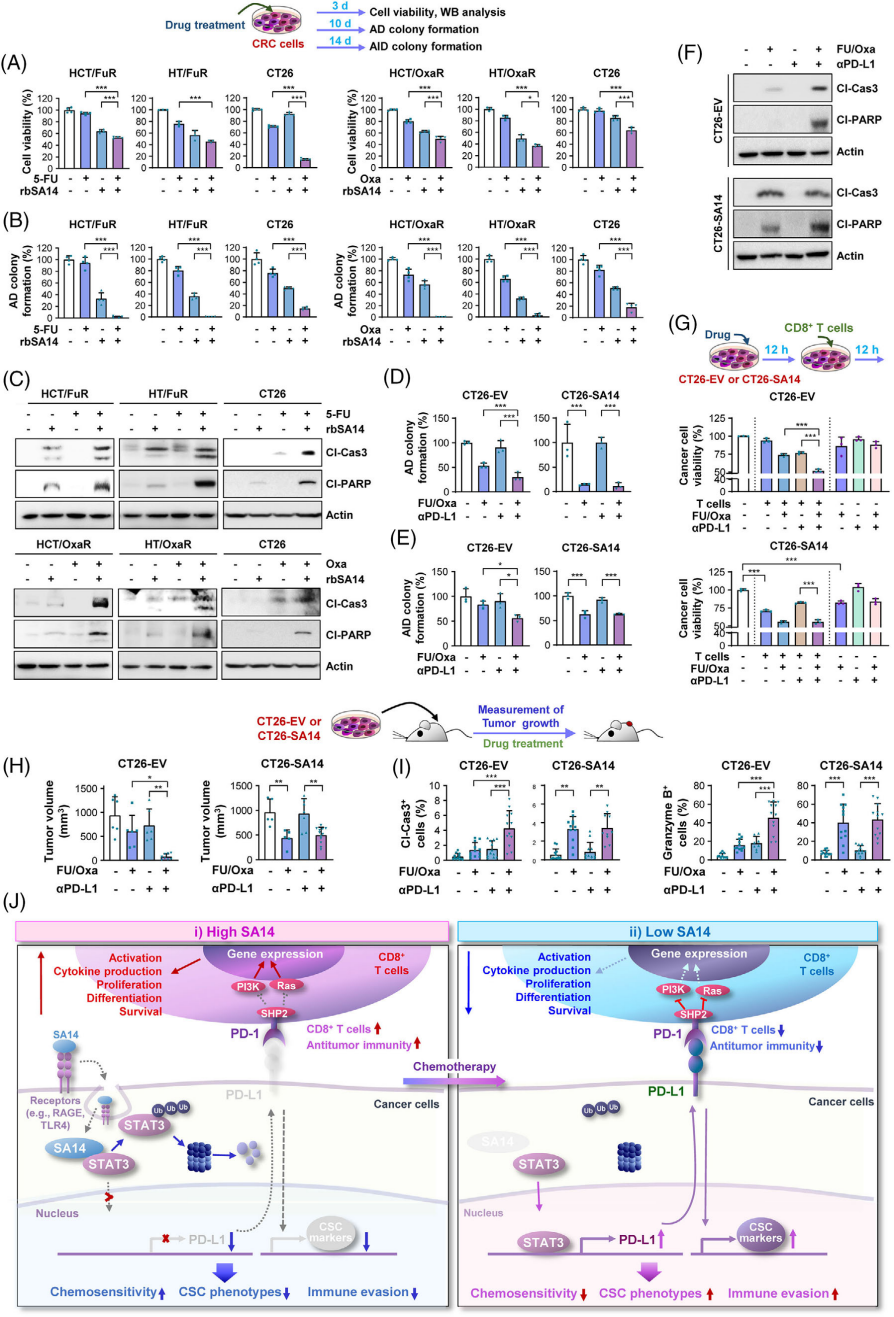
SA14 regulates the antitumour effect of the anti‐programmed death‐ligand 1 (PD‐L1) antibody and chemotherapy. (A–C) Regulation of the effect of a chemotherapeutic agent [5‐fluorouracil (5‐FU) or oxaliplatin (Oxa); 10 μM 5‐FU and 5 μM Oxa for cell viability of HCT116 and HT‐29 cells; 2 μM 5‐FU and 1 μM Oxa for cell viability of CT26 cells; 5 μM 5‐FU and 1 μM Oxa for colony formation of HCT116 and HT‐29 cells; 0.25 μM 5‐FU and 0.5 μM Oxa for colony formation of CT26 cells; 5 μM 5‐FU and 2 μM Oxa for apoptosis] on cell viability (A), anchorage‐dependent (AD) colony formation (B), and apoptosis (C) by treatment with recombinant SA14 protein (rbSA14; 1 μg/ml for cell viability and apoptosis; 0.5 μg/ml for colony formation). (D–G) Regulation of the inhibitory effects of anti‐PD‐L1 antibody (D–F: 2 μg; G: 10 μg), either alone or in combination with 5‐FU and Oxa (FU/Oxa; D and E: 0.1 μM 5‐FU and 0.5 μM Oxa; F: 2 μM 5‐FU and 4 μM Oxa; G: 1 μM 5‐FU and 2 μM Oxa), on the AD (D) and anchorage‐independent (AID) (E) colony formation, apoptosis (F) and tumour cell killing activity of T cells (G) by SA14 overexpression in CT26 cells. (H and I) Regulation of the inhibitory effects of anti‐PD‐L1 antibody (αPD‐L1, 100 μg, twice a week), either alone or in combination with FU/Oxa (50 mg/kg 5‐FU and 6 mg/kg Oxa in combination, once a week), on tumour growth (H) and tumoural expression of cleaved caspase‐3 and granzyme B (I) by SA14 overexpression in CT26 cells (CT26‐EV, Con: *n* = 6, FU/Oxa: *n* = 6. αPD‐L1: *n* = 6, FU/Oxa + αPD‐L1: *n* = 8; CT26‐SA14, Con: *n* = 5, FU/Oxa: *n* = 5. αPD‐L1: *n* = 7, FU/Oxa + αPD‐L1: *n* = 12). (J) Schematic diagram of the association of SA14 with chemoresistance. In chemo‐naïve cells, the direct association of SA14 with signal transducer and activator of transcription 3 (STAT3) causes the proteasomal degradation of the latter. Chronic exposure to chemotherapeutic agents causes downregulation of SA14, stabilisation and activation of STAT3, and increases in the transcription of several STAT3 target genes, including PD‐L1 and stemness markers, leading to the acquisition of cancer stem cells (CSC)‐like phenotypes and chemoresistance. The bars represent the mean ± standard deviation (SD); *p < .05, **p < .01 and ***p < .001, determined through one‐way analysis of variance (ANOVA) with Tukey's post hoc test (A, B, D, E, G, H, I). Cl‐Cas3: cleaved caspase‐3; Cl‐PARP: cleaved poly‐(ADP‐ribose) polymerase; EV: empty vector; HCT: HCT116, HT: HT‐29

Extracellular S100 proteins were found to bind to RAGE or other cellular receptors and to be internalised.^52^ Indeed, treatment with recombinant SA14 protein (rbSA14) efficiently increased intracellular SA14 and reduced STAT3 and PD‐L1 expression (Figure S4B). To translate our findings to therapeutic applications, we investigated the antitumor effects of rbSA14, either alone or in combination with either 5‐FU or Oxa. Compared with single or vehicle treatments, combined treatment with rbSA14 and a chemotherapeutic agent (5‐FU or Oxa) showed significantly greater inhibitory effects on viability (Figure 7A) and AD colony formation (Figure 7B) of HCT/FuR, HCT/OxaR and CT26 cells. Moreover, the combined treatment markedly induced apoptosis in HCT/FuR, HCT/OxaR and CT26 cells (Figure 7C).

Based on the SA14‐mediated regulation of PD‐L1 expression, we speculated that SA14 expression could predict the antitumour effect of anti‐PD‐L1 Abs. Thus, we examined the therapeutic activity of an anti‐PD‐L1 Ab in vitro and in vivo, either alone or in combination with FU/Oxa, in chemoresistant CRC cells carrying enforced SA14 expression. Because HCT/FuR‐SA14, HCT/OxaR‐SA14 and CT26‐SA14 cells exhibited similar behaviours, we used only CT26‐EV and CT26‐SA14 cells that could be analysed in immunocompetent mice. We found that treatment with anti‐PD‐L1 Ab alone showed minimal effects on AD (Figure 7D) and AID (Figure 7E) colony formation of both CT26‐EV and CT26‐SA14 cells. FU/Oxa treatment suppressed the colony‐forming capacities of these cells, with greater inhibitory activities in CT26‐SA14 cells than in CT26‐EV cells (Figure 7D,E). Notably, CT26‐EV cells, but not CT26‐SA14 cells, showed a significantly greater reduction in colony formation capacities after treatment with FU/Oxa and anti‐PD‐L1 Ab in combination compared to each single drug treatment (Figure 7D,E). Moreover, the combined treatment induced apoptotic activities in CT26‐EV cells, while treatment with anti‐PD‐L1 Ab minimally enhanced FU/Oxa‐induced apoptosis in CT26‐SA14 cells (Figure 7F). Similarly, T cells showed greater cytotoxicity against CT26‐SA14 cells than CT26‐EV cells (Figure 7G). CT26‐EV cells revealed an enhanced response towards T‐cell‐induced cytotoxicity after prior combinatorial treatment with FU/Oxa and anti‐PD‐L1 Ab, while anti‐PD‐L1 Ab did not further enhance the cytotoxicity of FU/Oxa against CT26‐SA14 cells (Figure 7G). Consistently, FU/Oxa treatment significantly reduced the volume of allograft tumours of CT26‐SA14 cells (Figure 7H). Compared with the single treatment, combinatorial treatment with FU/Oxa and anti‐PD‐L1 Ab significantly enhanced growth‐inhibitory effects only on CT26‐EV allograft tumours but not CT26‐SA14 tumours. IHC examination of the tumours indicated that FU/Oxa treatment significantly elevated caspase‐3 cleavage and granzyme B expression in CT26‐SA14 allograft tumours (Figure 7I, S4C). In addition, the combination treatment induced great increases in cleaved caspase‐3 and granzyme B compared with the other treatments in CT26‐EV allograft tumours, but these levels were not further enhanced in CT26‐SA14 allograft tumours (Figure 7I and S4C).

In general, ICIs are known to induce adverse effects (AEs), such as pruritus, diarrhoea, hepatitis, arthralgia, fatigue, thyroid dysfunction, rash and fever.^42^ In addition, the combination of conventional therapy (chemotherapy, targeted therapy, or their combination) with an ICI showed increased levels of AEs and toxicity compared with either therapy alone.^53^, ^54^ Therefore, it is possible that combining anti‐PD‐L1 immunotherapy with chemotherapy might have increased AEs in CRC. Treatment with 5‐FU and Oxa in combination altered the levels of several cytokines and chemokines, which might be associated with chemotherapy‐induced AEs such as lymphotoxicity, nephrotoxicity, cognitive impairment and neuropathic pain.^55^, ^56^, ^57^, ^58^ We then monitored the potential toxicities of the 5‐FU/Oxa and anti‐PD‐L1 mAb combination by analysing hepatic and renal toxicity in mice. We found no statistically significant variation in serum levels of AST (a marker for hepatic toxicity), ALT (a marker for hepatic toxicity), BUN (a marker for renal toxicity) and creatinine (a marker for renal toxicity) in the mice treated with vehicle and those treated with 5‐FU/Oxa or anti‐PD‐L1 Ab, either alone or in combination. We next analysed the cytokine/chemokine expression profile in serum from these mice using a commercially available cytokine array. We observed that several cytokines, including IL‐4, IL‐7, IL‐10, CXCL2, CCL1, CCL11, CCL12, IFN‐γ, G‐CSF, GM‐CSF and TREM‐1, displayed more than twofold increases in the FU/Oxa‐treated group compared with those in the control group. However, most of these cytokines/chemokines were either decreased or unchanged in mice treated with the 5‐FU/Oxa and anti‐PD‐L1 mAb combination compared to those treated with 5‐FU/Oxa or anti‐PD‐L1 mAb. Therefore, it is likely that the 5‐FU/Oxa and anti‐PD‐L1 mAb combination exerted no overt toxicity compared to the 5‐FU/Oxa or anti‐PD‐L1 mAb treatments (Figure S5A,B). Several clinical trials are underway to investigate the effectiveness of anti‐PD‐L1 immunotherapy in combination with standard therapeutic regimens [e.g., chemotherapy (FOLFOX or FOLFOXIRI) plus bevacizumab] in metastatic colorectal cancer.^42^ Hence, the AEs of combined chemotherapy and anti‐PD‐L1 immunotherapy would be defined after completion of the ongoing clinical trials. These results collectively suggest that SA14 inhibits the acquisition of immune‐suppressive activities, CSC‐like properties and chemoresistance of CRC cells, at least in part, by suppressing STAT3‐mediated PD‐L1 expression. In addition, SA14 can be a potential marker for predicting the antitumour response of PD‐L1 blockade and chemotherapy in CRC, and combination treatment can be a safe antitumour therapeutic strategy.

## 4 DISCUSSION

The identification and characterisation of cell populations involved in cancer development and progression, comprehending their biology, and developing targeting strategies towards such populations are logical approaches for anticancer therapy. The current study sought to elucidate the involvement of PD‐L1 in the functional features of CSCs,^14^ to understand the regulatory mechanism of PD‐L1 expression in CRC, and to develop an effective strategy to target patients with PD‐L1^high^ CRC. We show herein that SA14 expression is significantly downregulated in PD‐L1^high^ CRC subpopulations, enabling them to have CSC‐like phenotypes and immune‐suppressive capacities. In our model, SA14 is internalised through ligation to cell surface receptors, interacts with STAT3, induces its proteasome‐mediated degradation, and suppresses STAT3‐mediated *CD274* expression, causing ablation of CSC‐like phenotypes and stimulation of CD8^+^ T‐cell‐mediated cytotoxicity, which ultimately restores chemosensitivity and suppresses tumour growth (Figure 7J‐i). Our results specifically emphasise that PD‐L1^high^ CRC subpopulations achieve chemoresistance, CSC‐like phenotypes and immune evasion through loss of SA14 expression (Figure 7J‐ii). We finally show that pharmacological ablation of PD‐L1 suppresses CSC‐like phenotypes and the immune‐suppressive capacity of SA14^low^CRC cells and restores their chemosensitivity, ultimately suppressing tumour growth. These results indicate that SA14 has a pivotal role in regulating the growth of CRC in hostile environments.

Despite the widespread use of 5‐FU and platinum compounds in various types of cancer, including CRC,^59^, ^60^, ^61^ their clinical application is limited due to primary and acquired chemoresistance.^62^ Since FOLFOX remains a main therapeutic option for the treatment of CRC, identifying and targeting the key pathways that cause chemoresistance is of importance. Studies have suggested that PD‐L1 is an important player not only in immune suppression but also in CSC phenotypes.^14^ In support of this notion, we also observed that PD‐L1^high^ CRC subpopulations exhibited functional features of CSCs, including chemoresistance, along with immune suppression. Consistently, CD133^high^CD44^high^ potential CSC populations within CRC cell lines and patient tumour tissues exhibited PD‐L1 expression. Moreover, the CRC sublines that were subjected to prolonged exposure to 5‐FU or Oxa for the establishment of a preclinical model of PD‐L1^high^ CRC displayed upregulation of PD‐L1 expression and CSC‐like properties, which were significantly abrogated by a PD‐L1 neutralising Ab. Therefore, it is likely that PD‐L1^high^ CRC cells represent a distinct CSC subpopulation.

An important question is how PD‐L1 expression is regulated in the CSC subpopulation within CRC. Inquiry of public data and our validation studies consistently showed downregulation of SA14 with an inverse correlation with PD‐L1 expression in established CRC sublines with resistance to various chemotherapeutics, PD‐L1^high^ or CD133^high^CD44^high^ CSC populations within CRC cell lines and patient tumour tissues. A recent study reported loss or reduced expression of *SA14* mRNA in CRC in association with high metastatic potential and poor prognostic outcome in patients with CRC.^28^ Previous studies have suggested that platinum‐based chemotherapy induced DNA methylation,^63^ and chemotherapy‐induced DNA damage response and DNA lesions modulated gene transcription.^64^ Therefore, epigenetic or transcriptional modulation might be associated with SA14 downregulation by chemotherapeutic agents. However, at this point, it is not clear whether repeated exposure to chemotherapy downregulated SA14 expression, causing acquired chemoresistance, or whether the SA14^low^ subpopulation carrying innate chemoresistance was enriched after the exposure. Nevertheless, our findings highlight the function of SA14 as a regulator of chemoresistance and immune surveillance and the potential utility of SA14 expression as a valuable biomarker in clinical studies, that is, loss of SA14 as an adverse prognostic factor in CRC patients and as a predictive biomarker for responsiveness to chemotherapy and anti‐PD‐L1‐based immunotherapy combination. Further clinical tumour biopsy studies are warranted to correlate SA14 expression levels and the response to ICIs, either alone or in combination with 5‐FU‐based chemotherapeutic regimens, in CRC patients.

The next important question is how SA14 regulates PD‐L1 expression. Our study identified a previously unrecognised function of SA14 as a negative regulator of STAT3, an important transcription factor for PD‐L1 expression. A previous study has shown that HDAC3 is involved in STAT3 acetylation and stabilisation.^65^, ^66^ We observed that SA14 decreased the acetylation of STAT3. Notably, SA14 directly bound to the STAT3 protein and induced its degradation via the ubiquitin‒proteasome pathway. A previous study demonstrated that the deubiquitinating enzyme USP28 directly interacts with STAT3 and increases its stability.^67^ Thus, regulation of STAT3 acetylation and recruitment of E3 ligase or deubiquitinating enzymes might be associated with the regulation of STAT3 protein stability by SA14. STAT3 is a master player in regulating immunity and CSC phenotypes.^68^ Blockade of STAT3 was found to enhance antitumour immunity by decreasing the levels of proinflammatory cytokines,^69^ and combinatorial use of a STAT3 inhibitor potentiated the antitumour efficacy of anti‐PD‐1/PD‐L1 Abs.^70^ Anti‐PD‐1/PD‐L1 immunotherapy has been adopted for patients with solid tumours with high mismatch repair deficiency/MSH.^71^ Therefore, the reduction in STAT3 by SA14 overexpression may also alleviate MSH in tumour cells and reduce the accumulation of neoantigens, which might blunt the antitumour effect of anti‐PD‐L1 immunotherapy. Several cell surface receptors have been known to interact with S100 proteins.^31^, ^52^ Further mechanistic studies are necessary to elucidate the mode of action of SA14, including the receptors that mediate SA14 endocytosis and the mechanism underlying SA14‐mediated destabilisation of the STAT3 protein.

Our study provides important translational implications. Given PD‐L1 overexpression and its functional role in a variety of histologically different epithelial malignancies, including CRC, SA14 may be associated with a variety of human malignancies. We have also shown reduced expression of SA14 during the progression of human colon cancer. SA14^high^ CRC cells are chemosensitive, and treatment with anti‐PD‐L1 Ab sensitises SA14^low^ CRC to chemotherapy. At this point, it is not clear whether repeated exposure to chemotherapy downregulated SA14 expression, causing acquired chemoresistance, or whether the SA14^low^ subpopulation carrying innate chemoresistance was enriched after the exposure. Nevertheless, our findings highlight the function of SA14 as a regulator of chemoresistance and immune surveillance and the potential of SA14 expression as valuable biomarkers for patients with CRC, such as loss of SA14 as an unfavourable prognostic factor and a predictive biomarker for responsiveness to chemotherapy and anti‐PD‐L1‐based immunotherapy combination. In a previous immunohistochemical study evaluating SA14 expression in colorectal tissues, tumours with more than 30% immunoreactivity were defined as being positive for SA14 expression.^28^ Therefore, although additional extensive investigation is needed, normal and abnormal ranges of SA14 expression can be determined based on the reported criteria. Further clinical tumour biopsy studies are warranted to address these points.

Although our study demonstrates the tumour‐suppressive effect of SA14, contradictory findings on the function of SA14 exist.^52^ Studies have shown different functions of SA14 depending on its location.^52^ Intracellular S100 protein was found to regulate protein phosphorylation, transcription factor activity, cell proliferation and differentiation by binding to effector proteins such as p53. In contrast, extracellularly secreted S100 proteins modulate a number of signal transduction pathways, including phosphoinositide 3‐kinase/Akt, mitogen‐activated protein kinases and Cdc42/Rac, by binding to a number of cell surface proteins. Therefore, the levels of SA14 exocytosis and the expression levels of SA14‐binding proteins and their downstream effectors might affect the functions of SA14 in different cancer types. Considering the potential role of SA14 in immune surveillance, the surrounding tumour microenvironment might also contribute to the cancer type‐dependent function of SA14. Cancer cells in organs constitutively exposed to various hazardous substances or microbiomes, such as oral, stomach, lung and CRCs, may have different responses to SA14 compared to cancer cells that are not in such microenvironments. Further studies are warranted to investigate the mechanisms responsible for the controversial function of SA14.

## 5 CONCLUSIONS

Our study identifies a previously unrecognised function of SA14 that regulates PD‐L1 expression, phenotypes of CSC and chemoresistance in CRC. Our data might help to design novel anti‐PD‐1/PD‐L1‐based immunotherapy in patients with CRC. Further investigations using clinical specimens are warranted to explore the clinical usefulness of SA14 as a predictive biomarker for anti‐PD‐1/PD‐L1‐based anticancer therapy, the detailed mechanisms underlying the regulation of SA14 expression, the potential side effects when combining chemotherapeutic agents with anti‐PD‐L1 in patients with CRC, and the efficacy of various pharmacological approaches that modulate SA14 expression as anticancer regimens.

## CONFLICT OF INTEREST

There are no potential conflicts of interest to declare.

## Supporting information

Supplementary material

## References

[1] Sung H , Ferlay J , Siegel RL , et al. Global cancer statistics 2020: GLOBOCAN estimates of incidence and mortality worldwide for 36 cancers in 185 countries. CA Cancer J Clin. 2021;71(3):209‐249. 10.3322/caac.21660 33538338

[2] Jung KW , Won YJ , Hong S , Kong HJ , Im JS , Seo HG . Prediction of cancer incidence and mortality in Korea, 2021. Cancer Res Treat. 2021;53(2):316‐322. 10.4143/crt.2021.290 33735558 PMC8053854

[3] Xie YH , Chen YX , Fang JY . Comprehensive review of targeted therapy for colorectal cancer. Signal Transduct Target Ther. 2020;5(1):22. 10.1038/s41392-020-0116-z 32296018 PMC7082344

[4] Lu W , Fu D , Kong X , et al. FOLFOX treatment response prediction in metastatic or recurrent colorectal cancer patients via machine learning algorithms. Cancer Med. 2020;9(4):1419‐1429. 10.1002/cam4.2786 31893575 PMC7013065

[5] Golshani G , Zhang Y . Advances in immunotherapy for colorectal cancer: a review. Therap Adv Gastroenterol. 2020;13:1756284820917527. 10.1177/1756284820917527 PMC7268115 32536977

[6] Pardini B , Kumar R , Naccarati A , et al. 5‐Fluorouracil‐based chemotherapy for colorectal cancer and MTHFR/MTRR genotypes. Br J Clin Pharmacol. 2011;72(1):162‐163. 10.1111/j.1365-2125.2010.03892.x 21204909 PMC3141199

[7] Overman MJ , Ernstoff MS , Morse MA . Where we stand with immunotherapy in colorectal cancer: deficient mismatch repair, proficient mismatch repair, and toxicity management. Am Soc Clin Oncol Educ Book. 2018;38:239‐247. 10.1200/edbk_200821 30231358

[8] Voelker R . Immunotherapy is now first‐line therapy for some colorectal cancers. JAMA. 2020;324(5):433. 10.1001/jama.2020.13299 32749477

[9] Le DT , Uram JN , Wang H , et al. PD‐1 blockade in tumors with mismatch‐repair deficiency. N Engl J Med. 2015;372(26):2509‐2520. 10.1056/NEJMoa1500596 26028255 PMC4481136

[10] Salas‐Benito D , Perez‐Gracia JL , Ponz‐Sarvise M , et al. Paradigms on immunotherapy combinations with chemotherapy. Cancer Discov. 2021;11(6):1353‐1367. 10.1158/2159-8290.CD-20-1312 33712487

[11] Bagnyukova TV , Serebriiskii IG , Zhou Y , Hopper‐Borge EA , Golemis EA , Astsaturov I . Chemotherapy and signaling: how can targeted therapies supercharge cytotoxic agents? Cancer Biol Ther. 2010;10(9):839‐853. 10.4161/cbt.10.9.13738 20935499 PMC3012138

[12] Galluzzi L , Buque A , Kepp O , Zitvogel L , Kroemer G . Immunological effects of conventional chemotherapy and targeted anticancer agents. Cancer Cell. 2015;28(6):690‐714. 10.1016/j.ccell.2015.10.012 26678337

[13] Dosset M , Vargas TR , Lagrange A , et al. PD‐1/PD‐L1 pathway: an adaptive immune resistance mechanism to immunogenic chemotherapy in colorectal cancer. Oncoimmunology. 2018;7(6):e1433981. 10.1080/2162402X.2018.1433981 29872568 PMC5980491

[14] Escors D , Gato‐Canas M , Zuazo M , et al. The intracellular signalosome of PD‐L1 in cancer cells. Signal Transduct Target Ther. 2018;3:26. 10.1038/s41392-018-0022-9 30275987 PMC6160488

[15] Dong P , Xiong Y , Yue J , Hanley SJB , Watari H . Tumor‐intrinsic PD‐L1 signaling in cancer initiation, development and treatment: beyond immune evasion. Front Oncol. 2018;8:386. 10.3389/fonc.2018.00386 30283733 PMC6156376

[16] Wei F , Zhang T , Deng SC , et al. PD‐L1 promotes colorectal cancer stem cell expansion by activating HMGA1‐dependent signaling pathways. Cancer Lett. 2019;450:1‐13. 10.1016/j.canlet.2019.02.022 30776481

[17] Zahra P , Abbas Pirpour T , Behzad M , et al. The potential role of Nrf2‐PD‐L1 axis in promoting of oxaliplatin resistance in colon cancer cells. Res Square. 2021. 10.21203/rs.3.rs-58926/v1

[18] Zerdes I , Matikas A , Bergh J , Rassidakis GZ , Foukakis T . Genetic, transcriptional and post‐translational regulation of the programmed death protein ligand 1 in cancer: biology and clinical correlations. Oncogene. 2018;37(34):4639‐4661. 10.1038/s41388-018-0303-3 29765155 PMC6107481

[19] Marenholz I , Heizmann CW , Fritz G . S100 proteins in mouse and man: from evolution to function and pathology (including an update of the nomenclature). Biochem Biophys Res Commun. 2004;322(4):1111‐1122. 10.1016/j.bbrc.2004.07.096 15336958

[20] Pleger ST , Most P , Katus HA . S100 proteins: a missing piece in the puzzle of heart failure? Cardiovasc Res. 2007;75(1):1‐2. 10.1016/j.cardiores.2007.05.009 17531210

[21] Donato R . Intracellular and extracellular roles of S100 proteins. Microsc Res Tech. 2003;60(6):540‐551. 10.1002/jemt.10296 12645002

[22] Bianchi R , Giambanco I , Donato R . S100B/RAGE‐dependent activation of microglia via NF‐kappaB and AP‐1 co‐regulation of COX‐2 expression by S100B, IL‐1beta and TNF‐alpha. Neurobiol Aging. 2010;31(4):665‐677. 10.1016/j.neurobiolaging.2008.05.017 18599158

[23] Hermani A , De Servi B , Medunjanin S , Tessier PA , Mayer D . S100A8 and S100A9 activate MAP kinase and NF‐kappaB signaling pathways and trigger translocation of RAGE in human prostate cancer cells. Exp Cell Res. 2006;312(2):184‐197. 10.1016/j.yexcr.2005.10.013 16297907

[24] Leclerc E , Fritz G , Weibel M , Heizmann CW , Galichet A . S100B and S100A6 differentially modulate cell survival by interacting with distinct RAGE (receptor for advanced glycation end products) immunoglobulin domains. J Biol Chem. 2007;282(43):31317‐31331. 10.1074/jbc.M703951200 17726019

[25] Pietas A , Schluns K , Marenholz I , Schafer BW , Heizmann CW , Petersen I . Molecular cloning and characterization of the human S100A14 gene encoding a novel member of the S100 family. Genomics. 2002;79(4):513‐522. 10.1006/geno.2002.6744 11944983

[26] Basnet S , Sharma S , Costea DE , Sapkota D . Expression profile and functional role of S100A14 in human cancer. Oncotarget. 2019;10(31):2996‐3012. 10.18632/oncotarget.26861 31105881 PMC6508202

[27] Diamantopoulou A , Mantas D , Kostakis ID , et al. A clinicopathological analysis of S100A14 expression in colorectal cancer. In Vivo. 2020;34(1):321‐330. 10.21873/invivo.11777 31882495 PMC6984099

[28] Wang HY , Zhang JY , Cui JT , et al. Expression status of S100A14 and S100A4 correlates with metastatic potential and clinical outcome in colorectal cancer after surgery. Oncol Rep. 2010;23(1):45‐52. 19956863

[29] Sapkota D , Bruland O , Costea DE , Haugen H , Vasstrand EN , Ibrahim SO . S100A14 regulates the invasive potential of oral squamous cell carcinoma derived cell‐lines in vitro by modulating expression of matrix metalloproteinases, MMP1 and MMP9. Eur J Cancer. 2011;47(4):600‐610. 10.1016/j.ejca.2010.10.012 21074410

[30] Sapkota D , Costea DE , Blo M , et al. S100A14 inhibits proliferation of oral carcinoma derived cells through G1‐arrest. Oral Oncol. 2012;48(3):219‐225. 10.1016/j.oraloncology.2011.10.001 22032898

[31] Xu C , Chen H , Wang X , et al. S100A14, a member of the EF‐hand calcium‐binding proteins, is overexpressed in breast cancer and acts as a modulator of HER2 signaling. J Biol Chem. 2014;289(2):827‐837. 10.1074/jbc.M113.469718 24285542 PMC3887208

[32] Zhu H , Gao W , Li X , et al. S100A14 promotes progression and gemcitabine resistance in pancreatic cancer. Pancreatology. 2021;21(3):589‐598. 10.1016/j.pan.2021.01.011 33579599

[33] Chen H , Yuan Y , Zhang C , et al. Involvement of S100A14 protein in cell invasion by affecting expression and function of matrix metalloproteinase (MMP)‐2 via p53‐dependent transcriptional regulation. J Biol Chem. 2012;287(21):17109‐17119. 10.1074/jbc.M111.326975 22451655 PMC3366841

[34] Chen H , Ma J , Sunkel B , et al. S100A14: novel modulator of terminal differentiation in esophageal cancer. Mol Cancer Res. 2013;11(12):1542‐1553. 10.1158/1541-7786.MCR-13-0317 24107296

[35] Zhu M , Wang H , Cui J , et al. Calcium‐binding protein S100A14 induces differentiation and suppresses metastasis in gastric cancer. Cell Death Dis. 2017;8(7):e2938. 10.1038/cddis.2017.297 28726786 PMC5550849

[36] Colon K , Speicher DW , Smith P , et al. S100A14 is increased in activated NK cells and plasma of HIV‐exposed seronegative people who inject drugs and promotes monocyte‐NK crosstalk. J Acquir Immune Defic Syndr. 2019;80(2):234‐241. 10.1097/QAI.0000000000001911 30422902 PMC6331283

[37] Lee HJ , Min HY , Yong YS , et al. A novel C‐terminal heat shock protein 90 inhibitor that overcomes STAT3‐Wnt‐beta‐catenin signaling‐mediated drug resistance and adverse effects. Theranostics. 2022;12(1):105‐125. 10.7150/thno.63788 34987637 PMC8690924

[38] Cho J , Min HY , Lee HJ , et al. RGS2‐mediated translational control mediates cancer cell dormancy and tumor relapse. J Clin Invest. 2021;131(1):e136779. 10.1172/JCI136779 PMC7773398 33393490

[39] Labun K , Montague TG , Krause M , Torres Cleuren YN , Tjeldnes H , Valen E . CHOPCHOP v3: expanding the CRISPR web toolbox beyond genome editing. Nucleic Acids Res. 2019;47(W1):W171‐W174. 10.1093/nar/gkz365 31106371 PMC6602426

[40] Oh SH , Woo JK , Yazici YD , et al. Structural basis for depletion of heat shock protein 90 client proteins by deguelin. J Natl Cancer Inst. 2007;99(12):949‐961. 10.1093/jnci/djm007 17565155

[41] Sarangthem V , Kim Y , Singh TD , et al. Multivalent targeting based delivery of therapeutic peptide using AP1‐ELP carrier for effective cancer therapy. Theranostics. 2016;6(12):2235‐2249. 10.7150/thno.16425 27924160 PMC5135405

[42] Wu Y , Chen W , Xu ZP , Gu W . PD‐L1 distribution and perspective for cancer immunotherapy‐blockade, knockdown, or inhibition. Front Immunol. 2019;10:2022. 10.3389/fimmu.2019.02022 31507611 PMC6718566

[43] Shi G , Yang Q , Zhang Y , et al. Modulating the tumor microenvironment via oncolytic viruses and CSF‐1R inhibition synergistically enhances anti‐PD‐1 immunotherapy. Mol Ther. 2019;27(1):244‐260. 10.1016/j.ymthe.2018.11.010 30527756 PMC6319306

[44] Lau J , Cheung J , Navarro A , et al. Tumour and host cell PD‐L1 is required to mediate suppression of anti‐tumour immunity in mice. Nat Commun. 2017;8:14572. 10.1038/ncomms14572 28220772 PMC5321797

[45] Zhou HM , Zhang JG , Zhang X , Li Q . Targeting cancer stem cells for reversing therapy resistance: mechanism, signaling, and prospective agents. Signal Transduct Target Ther. 2021;6(1):62. 10.1038/s41392-020-00430-1 33589595 PMC7884707

[46] Yang L , Shi P , Zhao G , et al. Targeting cancer stem cell pathways for cancer therapy. Signal Transduct Target Ther. 2020;5(1):8. 10.1038/s41392-020-0110-5 32296030 PMC7005297

[47] Qian J , Wang C , Wang B , et al. The IFN‐gamma/PD‐L1 axis between T cells and tumor microenvironment: hints for glioma anti‐PD‐1/PD‐L1 therapy. J Neuroinflamm. 2018;15(1):290. 10.1186/s12974-018-1330-2 PMC6192101 30333036

[48] Shibue T , Weinberg RA . EMT, CSCs, and drug resistance: the mechanistic link and clinical implications. Nat Rev Clin Oncol. 2017;14(10):611‐629. 10.1038/nrclinonc.2017.44 28397828 PMC5720366

[49] Qin S , Xu L , Yi M , Yu S , Wu K , Luo S . Novel immune checkpoint targets: moving beyond PD‐1 and CTLA‐4. Mol Cancer. 2019;18(1):155. 10.1186/s12943-019-1091-2 31690319 PMC6833286

[50] Schust J , Sperl B , Hollis A , Mayer TU , Berg T . Stattic: a small‐molecule inhibitor of STAT3 activation and dimerization. Chem Biol. 2006;13(11):1235‐1242. 10.1016/j.chembiol.2006.09.018 17114005

[51] Al‐Ismaeel Q , Neal CP , Al‐Mahmoodi H , et al. ZEB1 and IL‐6/11‐STAT3 signalling cooperate to define invasive potential of pancreatic cancer cells via differential regulation of the expression of S100 proteins. Br J Cancer. 2019;121(1):65‐75. 10.1038/s41416-019-0483-9 31123345 PMC6738112

[52] Donato R , Cannon BR , Sorci G , et al. Functions of S100 proteins. Curr Mol Med. 2013;13(1):24‐57. 22834835 PMC3707951

[53] Zhou C , Cheng X , Tu S . Current status and future perspective of immune checkpoint inhibitors in colorectal cancer. Cancer Lett. 2021;521:119‐129. 10.1016/j.canlet.2021.07.023 34464671

[54] Xu C , Chen YP , Du XJ , et al. Comparative safety of immune checkpoint inhibitors in cancer: systematic review and network meta‐analysis. BMJ. 2018;363:k4226. 10.1136/bmj.k4226 30409774 PMC6222274

[55] Grasselly C , Denis M , Bourguignon A , et al. The antitumor activity of combinations of cytotoxic chemotherapy and immune checkpoint inhibitors is model‐dependent. Front Immunol. 2018;9:2100. 10.3389/fimmu.2018.02100 30356816 PMC6190749

[56] Malyszko J , Kozlowska K , Kozlowski L , Malyszko J . Nephrotoxicity of anticancer treatment. Nephrol Dial Transplant. 2017;32(6):924‐936. 10.1093/ndt/gfw338 28339935

[57] Cheung YT , Lim SR , Ho HK , Chan A . Cytokines as mediators of chemotherapy‐associated cognitive changes: current evidence, limitations and directions for future research. PloS ONE. 2013;8(12):e81234. 10.1371/journal.pone.0081234 24339912 PMC3855252

[58] Brandolini L , d'Angelo M , Antonosante A , Allegretti M , Cimini A . Chemokine signaling in chemotherapy‐induced neuropathic pain. Int J Mol Sci. 2019;20(12). 10.3390/ijms20122904 PMC6627296 31197114

[59] Longley DB , Harkin DP , Johnston PG . 5‐Fluorouracil: mechanisms of action and clinical strategies. Nat Rev Cancer. 2003;3(5):330‐338. 10.1038/nrc1074 12724731

[60] Olaussen KA , Postel‐Vinay S . Predictors of chemotherapy efficacy in non‐small‐cell lung cancer: a challenging landscape. Ann Oncol. 2016;27(11):2004‐2016. 10.1093/annonc/mdw321 27502726

[61] Gaurav S , Durgadas A , Rajshree K , Ravindra KR . Oxaliplatin for colorectal cancer therapy: a review. Clin Cancer Drugs. 2018;5(1):13‐27. 10.2174/2212697;05666180905094942

[62] Zhang N , Yin Y , Xu SJ , Chen WS . 5‐Fluorouracil: mechanisms of resistance and reversal strategies. Molecules. 2008;13(8):1551‐1569. 18794772 10.3390/molecules13081551 PMC6244944

[63] Flanagan JM , Wilson A , Koo C , et al. Platinum‐based chemotherapy induces methylation changes in blood DNA associated with overall survival in patients with ovarian cancer. Clin Cancer Res. 2017;23(9):2213‐2222. 10.1158/1078-0432.CCR-16-1754 27663594

[64] Capozzo I , Iannelli F , Francia S , d'Adda di Fagagna F . Express or repress? The transcriptional dilemma of damaged chromatin. FEBS J. 2017;284(14):2133‐2147. 10.1111/febs.14048 28231404

[65] Zhuang S . Regulation of STAT signaling by acetylation. Cell Signal. 2013;25(9):1924‐1931. 10.1016/j.cellsig.2013.05.007 23707527 PMC4550442

[66] Lee SC , Min HY , Jung HJ , et al. Essential role of insulin‐like growth factor 2 in resistance to histone deacetylase inhibitors. Oncogene. 2016;35(42):5515‐5526. 10.1038/onc.2016.92 27086926 PMC5069101

[67] Li P , Huang Z , Wang J , Chen W , Huang J . Ubiquitin‐specific peptidase 28 enhances STAT3 signaling and promotes cell growth in non‐small‐cell lung cancer. Onco Targets Ther. 2019;12:1603‐1611. 10.2147/OTT.S194917 30881015 PMC6396656

[68] Hillmer EJ , Zhang H , Li HS , Watowich SS . STAT3 signaling in immunity. Cytokine Growth Factor Rev. 2016;31:1‐15. 10.1016/j.cytogfr.2016.05.001 27185365 PMC5050093

[69] Grivennikov SI , Greten FR , Karin M . Immunity, inflammation, and cancer. Cell. 2010;140(6):883‐899. 10.1016/j.cell.2010.01.025 20303878 PMC2866629

[70] Woessner R , McCoon P , Bell K , et al. Abstract A93: STAT3 inhibition enhances the activity of immune checkpoint inhibitors in murine syngeneic tumor models by creating a more immunogenic tumor microenvironment. Cancer Immunol Res. 2015;3(10 suppl):A93. 10.1158/2326-6074.Tumimm14-a93

[71] Zhao P , Li L , Jiang X , Li Q . Mismatch repair deficiency/microsatellite instability‐high as a predictor for anti‐PD‐1/PD‐L1 immunotherapy efficacy. J Hematol Oncol. 2019;12(1):54. 10.1186/s13045-019-0738-1 31151482 PMC6544911

