# Supplementary material for: S100A14: A novel negative regulator of cancer stemness and immune evasion by inhibiting STAT3‐mediated programmed death‐ligand 1 expression in colorectal cancer
Source: Clin Transl Med. 2022 Jul 20;12(7):e986. doi: 10.1002/ctm2.986 (PMC9299575; doi:10.1002/ctm2.986)
Supplement: Supplementary file 1 — Supplementary material [file CTM2-12-e986-s001.pdf]

Supplementary materials

S100A14: A novel negative regulator of cancer stemness and immune evasion by inhibiting STAT3-mediated programmed death-ligand 1 expression n colorectal cancer

Hye-Young Min, Jaebeom Cho, Jeong Yeon Sim, Hye-Jin Boo, Ji-Sun Lee, Seon-Boon Lee, Young-Jin Lee, Sung Joo Kim, Kyu-Pyo Kim, In-Ja Park, Seung-Mo Hong, Xue-Li Zhang, Zhi-Gang Zhang, Rang-Woon Park, Ho-Young Lee

Table S1. Antibodies used in this study.

Target	Vendor	Catalogue Number	Clone	Dilution ratio	Application
STAT3	Santa Cruz	sc-482	C-20	1:1,000 (WB)<br>1:200 (IP)	WB, IF, IP
STAT3	Cell signaling	9139	124H6	1:200 (IF)<br>1: 500 (IHC)	IF, IHC
S100A14	Proteintech	10489-1-AP		1:1,000 (WB)<br>1:200 (IF)<br>1:200 (IHC)	WB, IF, IHC
pSTAT3	Cell signaling	9145	D3A7	1:1,000	WB
Ac-STAT3	Cell signaling	2523		1:1,000	WB
Oct4	Abcam	ab-19857		1:1,000	WB
Nanog	Cell signaling	4903	D73G4	1:1,000	WB
Sox2	Abcam	97959		1:1,000	WB
cMyc	Santa Cruz	sc-40	9E10	1:1,000	WB
Cleaved caspase-3	Cell signaling	9661		1:1,000 (WB)<br>1:200 (IHC)	WB, IHC
Cleaved PARP	Cell signaling	9544		1:1,000	WB
PD-L1	Cell signaling	13684	E1L3N	1:1,000	WB
PD-L2	Cell signaling	82723	D7U8C	1:1,000	WB
His	Santa Cruz	sc-8036	H-3	1:1,000	WB
GST	Santa Cruz	sc-459	Z-5	1:1,000	WB
Purified anti-human CD274	Biolegend	329702	29E.2A3	1:100 / 1.5×10^6 cells (FACS, FC)<br>1:50 (IF, IHC)<br>0.5 µg (Blockade)	FACS, IF, IHC, Blockade
PE anti-mouse CD274	Biolegend	124307	10F.9G2	1:100 / 1.5×10^6 cells	FACS
Purified anti-mouse CD274	Biolegend	124302	10F.9G2	1:50	IF

Anti-mouse PD-L1	InVivoMAb	BE0101	10F.9G2	8 μg/mL (WB) 10 μg/mL (blockade)	WB, Blockade
FITC anti-human CD44	BD Pharmingen	555478	G44-2	1:100/ test (~ 10^7 cells)	FACS
PE anti-human CD133	Miltenyl Biotec	130-090-853	293C3	1:100/ test (~ 10^7 cells)	FACS
FITC anti-mouse CD44	Biolegend	103005	IM7	1:100 / 1.5×10^6 cells	FACS
PE anti-mouse CD133	Biolegend	141203	315-2C11	1:100 / 1.5×10^6 cells	FACS
PE anti-mouse IFN-γ	Biolegend	505807	XMG1.2	1:100 / 1.5×10^6 cells	FC
APC anti-mouse CD8a	BioGems	10112-80-100	2.43	1:200 / 1.5×10^6 cells	FC
PerCP/Cy5.5 anti-mouse CD3e	BioGems	05122-70-100	145-2C11	1:200 / 1.5×10^6 cells	FC
Granzyme B	R&D	AF-1865		1:200	IHC
Ubiquitin	Santa Cruz	sc-8017	P4D1	1:1,000	WB
Purified Mouse IgG2b, κ Isotype Ctrl Antibody	Biolegend	402201	27-35	1:100 / 1.5×10^6 cells	FC
HRP-conjugated goat anti-mouse igG	GeneTex	213111-01		1:5,000	WB
HRP-conjugated goat anti-rabbit igG	GeneTex	213110-01		1:5,000	WB
Alexa Fluor 594-conjugated donkey anti-rabbit igG	Thermo Fisher Scientific	A21207		1:1,000	IF
Alexa Fluor 633-conjugated goat anti-mouse igG	Thermo Fisher Scientific	A21052		1:200	FACS

Alexa Fluor 488-conjugated goat anti-mouse igG	Thermo Fisher Scientific	A11001	1:1,000	FACS
Horse Anti-Goat IgG (H+L), Biotinylated	Vector lab	BA-9500	1:1,000	IHC
Goat Anti-Rabbit IgG (H+L), Biotinylated	Vector lab	BA-1000	1:1,000	IHC
Horse anti-Mouse IgG (H+L) Biotinylated	Vector lab	BA-2000	1:1,000	IHC

1)Application - WB: Western blot analysis; IF: Immunofluorescence staining; IHC: Immunohistochemistry; IP: Immunoprecipitation; FACS: Fluorescence-Activated Cell Sorting, FC: Flow cytometry

Table S2. A list of probes that were used to extract gene expression levels from GEO datasets.

Dataset	Platform	Gene	Probe
GSE24551	GPL5175	*CD274*	3161082
		POU5F1	2948863
		NANOG	3576633
		SOX2	2654454
		MYC	3115504
		VIM	3236958
		SNAI2	3134511
		CDH2	3802602
		S100A14 (*SA14*)	4045665
GSE20916	GPL570	*S100A14* (*SA14*)	218677_at
GSE41258	GPL96		
GSE92921	GPL570	*S100A14* (*SA14*)	218677_at
		CD274	227458_at
		POU5F1	208286_x_at
		MYC	202431_s_at
GSE59501	GPL6244	*S100A14* (*SA14*)	7920297

Table S3. Primer sequences used in this study.

Gene	Forward sequence (5´-3´)	Reverse sequence (5´-3´)	Application
S100A14	TCACCAAAGGACCAGACACA	GCCCTCTCCACATCACTGAA	Real-time PCR
CD274	TGCCGACTACAAGCGAATTACTG	CTGCTTGTCCAGATGACTTCGG	Real-time PCR
CSTA	AAACTCAAGTTGTTGCTGGAACAAA	TTTGTCAACCTGGTATCCAGTAAG	Real-time PCR
RSPSY1	TAAGAGGCTGGCCACCAAAC	AGACAGTGCCAAAATCATGAGC	Real-time PCR
RSPSY2	GGAGAGGTTCTGTTCCGTCG	CCTCAGCTTGTGAGGACCTG	Real-time PCR
POU5F1	CTGGGTTGATCCTCGGACCT	CCATCGGAGTTGCTCTCCA	Real-time PCR
NANOG	TGGGATTTACAGGCGTGAGCCAC	AAGCAAAGCCTCCCAATCCCAAAC	Real-time PCR
SOX2	TACAGCATGTCCTACTCGCAG	GAGGAAGAGGTAACCACAGGG	Real-time PCR
MYC	CAGCTGCTTAGACGCTGGATT	GTAGAAATACGGCTGCACCGA	Real-time PCR
ACTB	GCGAGAAGATGACCCAGATC	GGATAGCACAGCCTGGATAG	Real-time PCR
S100a14	GGAGTTTGGAAGCTTCTGGGA	CGAGTAACAGGCCTCTCCAT	Real-time PCR
Cd274	TGCGGACTACAAGCGAATCACG	CTCAGCTTCTGGATAACCCTCG	Real-time PCR
Pou5f1	CTGGGTTGATCCTCGGACCT	CCATCGGAGTTGCTCTCCA	Real-time PCR
Nanog	GAACGCCTCATCAATGCCTGCA	GAATCAGGGCTGCCTTGAAGAG	Real-time PCR
Sox2	TACAGCATGTCCTACTCGCAG	GAGGAAGAGGTAACCACAGGG	Real-time PCR
Myc	TCGCTGCTGTCCTCCGAGTCC	GGTTTGCCTCTTCTCCACAGAC	Real-time PCR
Actb	CGCCACCAGTTCGCCATGGA	TACAGCCCGGGGAGCATCGT	Real-time PCR

Table S4. Clinicopathologic characteristics of colorectal cancers according to SA14 (S100A14) expression status

Clinicopathological characteristics		n	SA14 expression, n (%)		*P*-value
			Loss	intact	
Age (mean ± SD)		94	57.6 ± 9.7	54.6 ± 10.7	0.152
Tumor size (mean ± SD)		94	5.8 ± 2.1	5.8 ± 2.4	0.929
Location					0.038*
	Proximal	28	12 (42.9)	16 (57.1)	
	Distal	66	43 (65.2)	23 (34.8)	
Differentiation†					0.667
	Well / moderate	85	50 (58.8)	35 (41.2)	
	Poor / undifferentiated	5	3 (60.0)	2 (40.0)	
Depth of invasion					0.439
	pT1 & pT2	8	4 (50.0)	4 (50.0)	
	pT3 & pT4	86	51 (59.3)	35 (40.7)	
Lymphovascular invasion					0.007*
	Absent	60	29 (48.3)	31 (51.7)	
	Present	34	26 (76.5)	8 (23.5)	
Perineural invasion					0.416
	Absent	70	40 (57.1)	30 (42.9)	
	Present	24	15 (62.5)	9 (37.5)	
Lymph node metastasis					0.016*
	Absent	42	19 (45.2)	23 (54.8)	
	Present	52	36 (69.2)	16 (30.8)	
Distant metastasis					0.006*
	Absent	52	24 (46.2)	28 (53.8)	
	Present	42	31 (73.8)	11 (26.2)	

* Significant at $p < 0.05$

† Exculde 4 cases of mucinous carcinoma cases from analysis

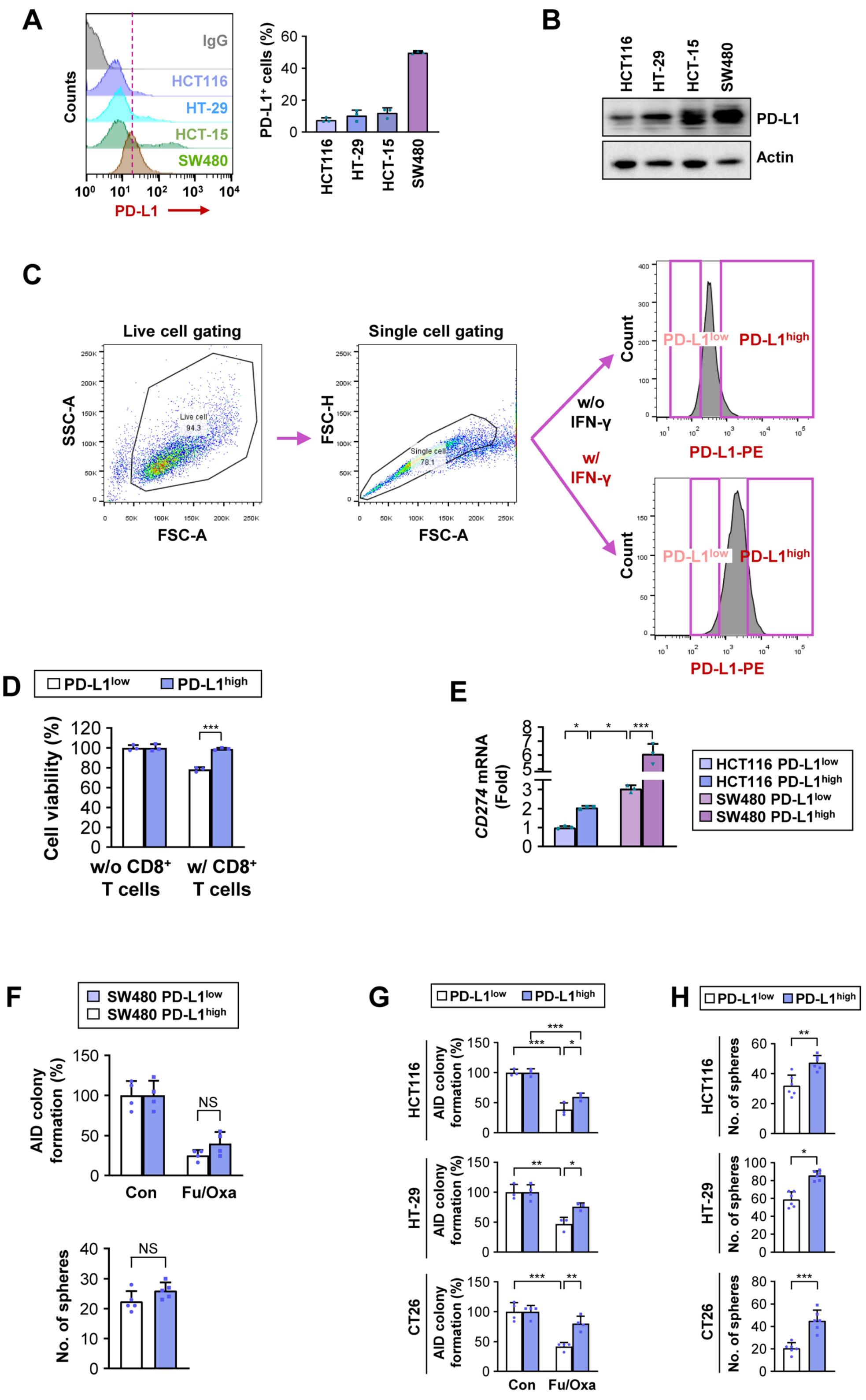

Figure S1. PD-L1 is differentially expressed in colorectal cancer cells, and the PD-L1^{high} population of CRC cells displays a decrease in tumor cell killing activity of CD8^{+} T cells, reduced chemosensitivity, and an increase in sphere formation capacity. (**A**) Flow

cytometry for determination of the level of PD-L1 expression on the cell membrane. (**B**) Determination of the level of PD-L1 expression by Western blot analysis. (**C**) Gating strategy for isolating PD-L1^{high} and PD-L1^{low} populations in CT26 cells with or without IFN-γ stimulation. (**D**) Crystal violet assay showing regulation of CD8^{+} T cell-mediated cytotoxicity in PD-L1^{high} and PD-L1^{low} populations of CT26 cells. (**E**) Real-time PCR analysis of *CD274* expression, which encodes the PD-L1 protein, in PD-L1^{high} and PD-L1^{low} populations of HCT116 (a PD-L1low expressor) and SW480 (a PD-L1high expressor) cells. (**F**) Changes in chemotherapy (0.1 μM 5-FU and 0.5 μM oxaliplatin in combination, FU/Oxa)-mediated regulation of anchorage-independent (AID) colony formation (top) and basal sphere formation (bottom) in PD-L1^{high} and PD-L1^{low} populations of SW480 cells. (**G**) Effect of a Fu/Oxa combination (0.1 μM 5-FU and 0.5 μM oxaliplatin in combination) on AID colony formation of PD-L1^{high} and PD-L1^{low} populations of HCT116, HT-29, and CT26 cells. The PD-L1^{high} and PD-L1^{low} populations were isolated after stimulation with IFN-γ (10 ng/mL) for 24 h. (**H**) Changes in sphere formation in PD-L1^{high} and PD-L1^{low} populations of HCT116, HT-29, and CT26 cells. The PD-L1^{high} and PD-L1^{low} populations were isolated after stimulation with IFN-γ (10 ng/mL) for 24 h. The bars represent the mean ± SD; *$p < 0.05$, **$p < 0.01$, and ***$p < 0.001$, as determined through one-way ANOVA with Tukey's post-hoc test (**D**, **E, F, G**) or a two-tailed Student's *t*-test by comparison with the indicated control (**F, H**).

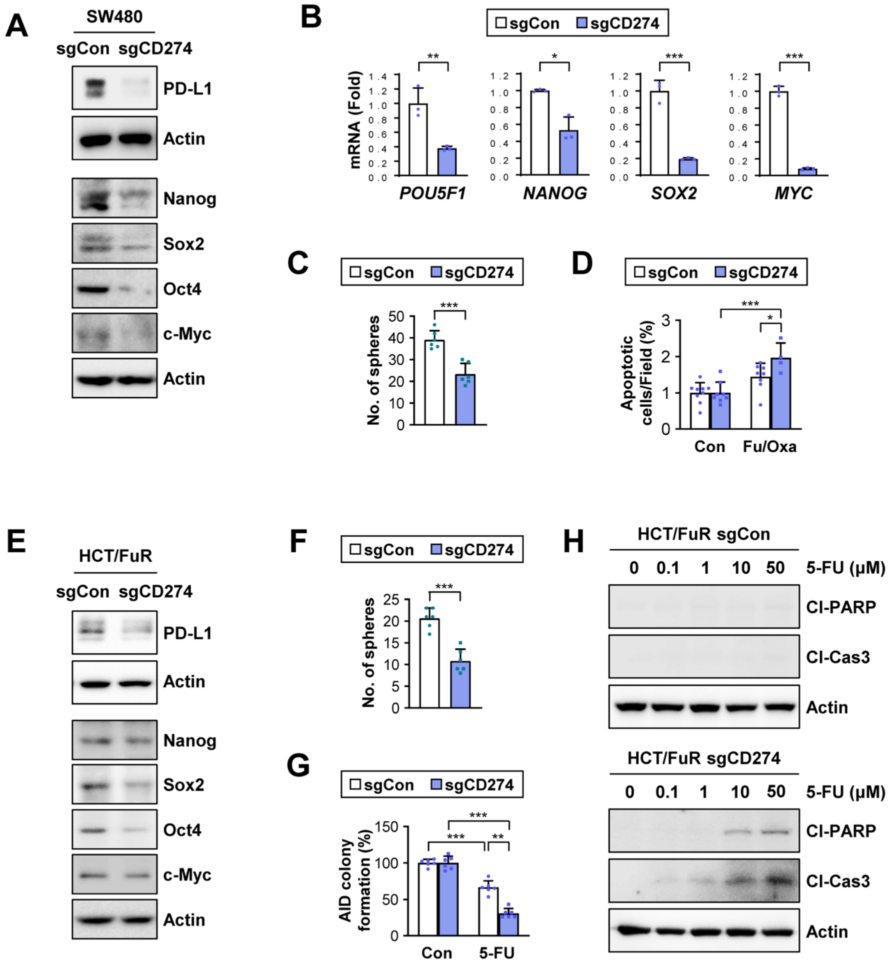

Figure S2. Silencing of PD-L1 expression sensitizes chemotherapy and reduces CSC-like properties in CRC cells. (**A-C**) Protein and mRNA expression levels of PD-L1 and CSC markers, determined by Western blot (WB) (**A**) and/or real-time PCR (**B**) analyses, and sphere-forming capacities (**C**) of SW480 cells transfected with a scrambled sgRNA (sgCon) or a sgCD274 (SW480 sgCD274). (**D**) Hoechst 33342 staining for apoptotic activities in SW480 sgCon and SW480 sgCD274 in the presence of 5-FU (0.2 μM) and oxa (4 μM) combination (FU/Oxa). (**E-H**) Analysis of HCT/FuR cells transfected with a scrambled sgRNA (sgCon) or a sgCD274 for the expression levels of PD-L1 and CSC marker proteins (**E**), sphere formation (**F**), anchorage-independent (AID) colony formation (**G**), and the expression levels of apoptosis marker proteins (**H**) in the absence or presence of 5-FU. The bars represent the mean ± SD; **p* < 0.05, ***p* < 0.01, and ****p* < 0.001, as determined through a two-tailed Student's *t*-test by comparison with the indicated control (**B, C, F**) or one-way ANOVA with Tukey's post-hoc test (**D, G**).

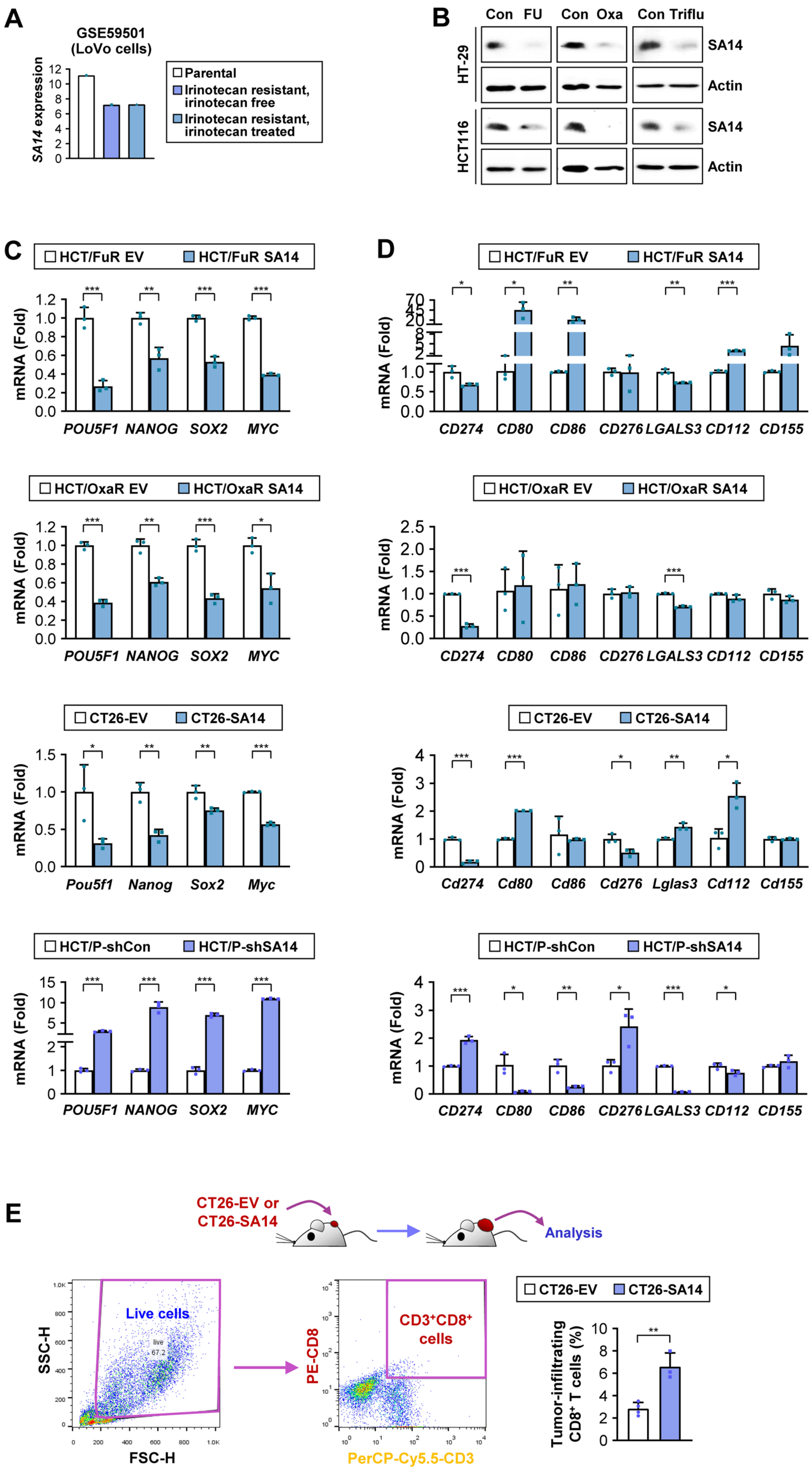
A
GSE59501
(LoVo cells)
SA14 expression
Parental
Irinotecan resistant, irinotecan free
Irinotecan resistant, irinotecan treated
B
Con FU Con Oxa Con Triflu
HT-29
HCT116
SA14
Actin
C
HCT/FuR EV
HCT/FuR SA14
mRNA (Fold)
POU5F1 NANOG SOX2 MYC
HCT/OxaR EV
HCT/OxaR SA14
CT26-EV
CT26-SA14
Pou5f1 Nanog Sox2 Myc
HCT/P-shCon
HCT/P-shSA14
D
CD274 CD80 CD86 CD276 LGALS3 CD112 CD155
Cd274 Cd80 Cd86 Cd276 Lglas3 Cd112 Cd155
E
CT26-EV or
CT26-SA14
Analysis
Live cells
SSC-H
FSC-H
PE-CD8
PerCP-Cy5.5-CD3
CD3⁺CD8⁺
cells
Tumor-infiltrating
CD8⁺ T cells (%)

Figure S3. SA14 expression is decreased by chemotherapy, and SA14 modulates stemness marker expression in CRC cells and the recruitment of CD8$^+$ T cells. (**A**) Analysis of a public GEO dataset (GSE59501) for the changes in S100A14 (SA14) expression in parental and irinotecan-resistant LoVo cells. (**B**) Western blot analysis of SA14 expression in HT-29 and HCT116 cells treated for 7 days with 5-FU (FU, 1 μM), oxaliplatin (Oxa, 1 μM), or trifluridine (Triflu, 5 μM). (**C, D**) The mRNA expression levels of CSC marker genes (**C**) and immune checkpoint-related genes (**D**) in HCT/FuR, HCT/OxaR, and CT26 cells transfected with mammalian expression vector carrying SA14 or empty vector (EV) and in HCT/P cells transfected with control (shCon) or SA14-specific shRNA (shSA14). (D) Gating strategy of flow cytometry for analyzing tumor-infiltrating CD8$^+$ T cells (left) and quantification of the tumor-infiltrating CD8$^+$ T cells (right) in CT26-EV and CT26-SA14 allograft tumors. The bars represent the mean ± SD; *p < 0.05, **p < 0.01, and ***p < 0.001, as determined by a two-tailed Student's t-test by comparison with the indicated control (**C-E**).

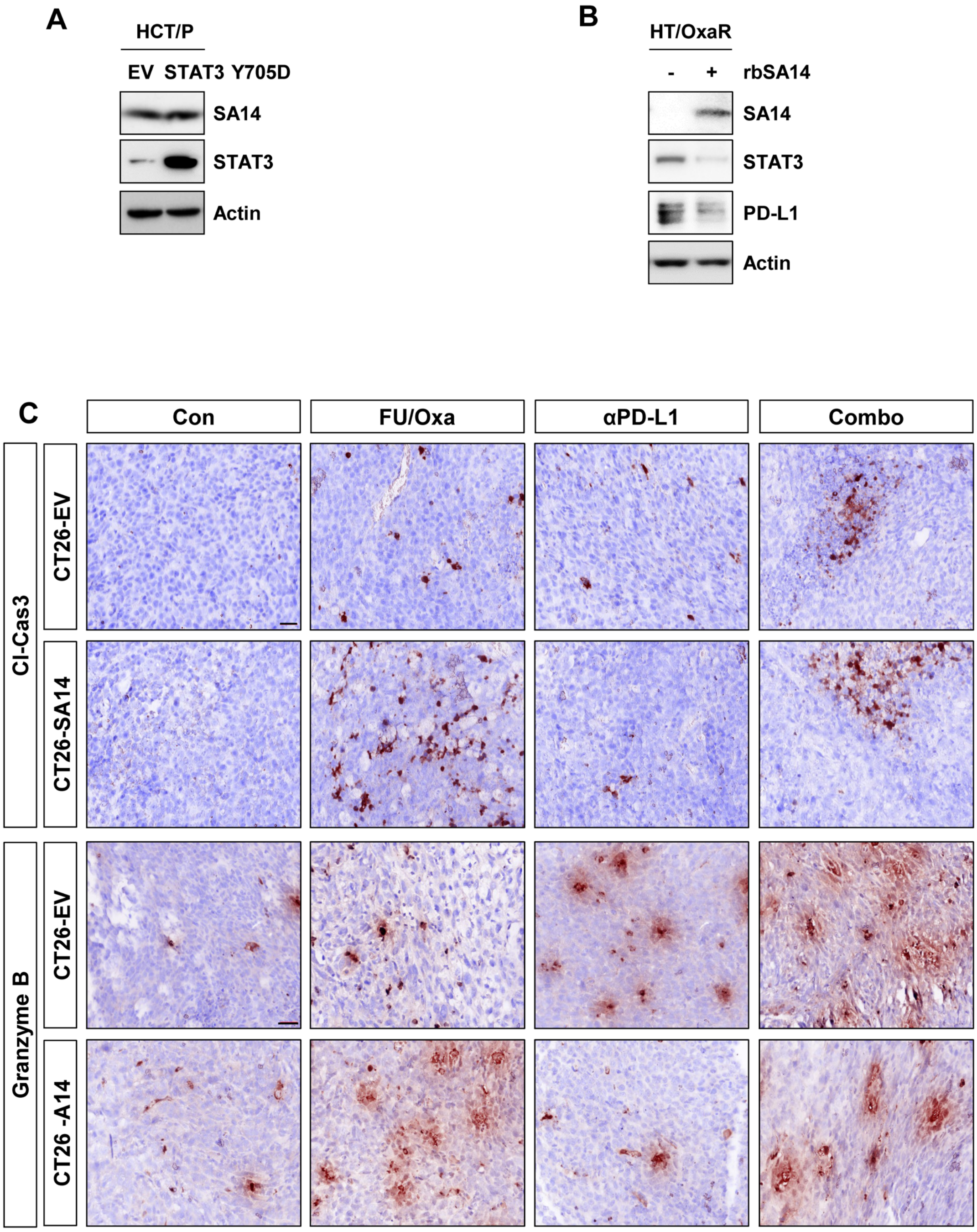

Figure S4. SA14 regulates STAT3 and PD-L1 in HCT/OxaR cells and tumoral level of cleaved caspase-3 and granzyme B expression in CT26 allograft tumors. (**A**) Western blot analysis showing regulation of S100A14 (SA14) in HCT/P cells with ectopic overexpression of the constitutive active form of STAT3 (STAT3 Y705D). (**B**) Western blot analysis showing regulation of SA14, STAT3, and PD-L1 in HT/OxaR cells treated with recombinant SA14 protein (rbSA14, 1 μg/mL). (**C**) Immunohistochemistry analysis showing changes in the level of cleaved caspase-3 (Cl-Cas3) and granzyme B in CT26-EV and CT26-SA14 allograft tumors treated with either chemotherapy (5-FU and oxaliplatin in combination, Fu/Oxa) or anti-PD-L1 antibody. The quantification analysis results are included in **Fig. 7I**. Scale bars: 20 μm. SA14: S100A14. Cl-Cas3: cleaved caspase-3.

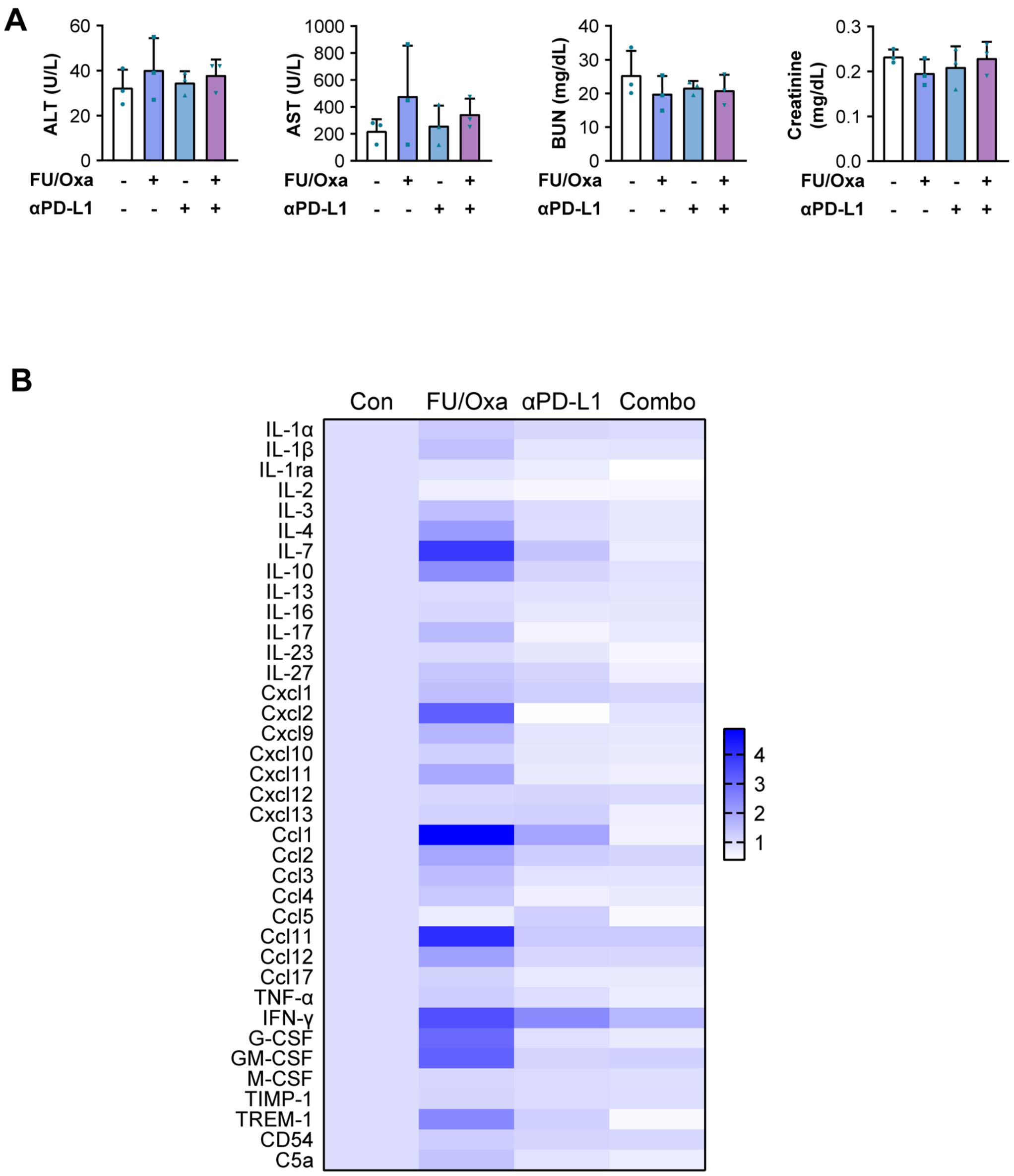

Figure S5. A combination of chemotherapy and anti-PD-L1 immunotherapy causes minimal toxicity in mice. (**A**) ALT, AST, BUN, and creatinine levels in serum from mice given FU/Oxa (50 mg/kg of 5-FU and 6 mg/kg of Oxa in combination, once a week) or anti-PD-L1 antibody (100 μg, twice a week), either alone or in combination. (**B**) Determination of various cytokines and chemokines in the serum of mice treated with the indicated drugs by using a cytokine array kit. The fold changes by comparison with the control group were depicted as a histogram. Densitometric analysis was performed using the ImageJ software.
